# Hepatitis B virus spliced variants are associated with an impaired response to interferon therapy

**DOI:** 10.1038/srep16459

**Published:** 2015-11-20

**Authors:** Jieliang Chen, Min Wu, Fan Wang, Wen Zhang, Wei Wang, Xiaonan Zhang, Jiming Zhang, Yinghui Liu, Yi Liu, Yanling Feng, Ye Zheng, Yunwen Hu, Zhenghong Yuan

**Affiliations:** 1 Key Laboratory of Medical Molecular Virology, School of Basic Medical Sciences, Shanghai Medical College of Fudan University, China; 2 Institutes of Medical Microbiology and Biomedical Sciences, Fudan University, Shanghai, China; 3 Shanghai Public Health Clinical Center, Shanghai Medical College of Fudan University, China; 4 Huashan Hospital affiliated to Fudan University, China

## Abstract

During hepatitis B virus (HBV) replication, spliced HBV genomes and splice-generated proteins have been widely described, however, their biological and clinical significance remains to be defined. Here, an elevation of the proportion of HBV spliced variants in the sera of patients with chronic hepatitis B (CHB) is shown to correlate with an impaired respond to interferon-α (IFN-α) therapy. Transfection of the constructs encoding the three most dominant species of spliced variants into cells or ectopic expression of the two major spliced protein including HBSP and N-terminal-truncated viral polymerase protein result in strong suppression of IFN-α signaling transduction, while mutation of the major splicing-related sites of HBV attenuates the viral anti-IFN activities in both cell and mouse models. These results have associated the productions of HBV spliced variants with the failure response to IFN therapy and illuminate a novel mechanism where spliced viral products are employed to resist IFN-mediated host defense.

Hepatitis B virus (HBV), which can cause chronic liver disease that may result in cirrhosis and hepatocellular carcinoma (HCC), is a DNA virus that has a 3.2 kB partially double-stranded, relaxed circular genome organized into overlapping open-reading frames from which viral genes are transcribed. The major HBV messenger RNAs (mRNA) synthesized from the HBV genome include: 3.5 kb (long/short) preC/pre-genomic (pg) RNA that encodes hepatitis B e antigen (HBeAg), core and polymerase (Pol) proteins, 2.4 kb and 2.1 kb sub-genomic RNAs that encode the HBV surface proteins (HBs), and a 0.9 kb mRNA that encodes the HBx protein. Through the 5′ stem-loop structure ε, the 3.5 kb pgRNA can also be packaged and reverse-transcribed by the viral polymerase into relaxed circular DNA to generate the wild-type HBV (wtHBV) particles for secretion into circulation^1^.

In addition to the unspliced mRNAs, a series of spliced (SP) HBV (spHBV) RNAs have been widely described in model systems and in HBV-infected livers^2^,^3^,^4^,^5^. The most frequently detected spHBV variant is a 2.2 kb molecule termed SP1, which is generated through the removal of a 1.3 kb intron from the pgRNA and accounts for up to 30% of pgRNAs^3^,^4^,^5^,^6^,^7^,^8^. spHBV RNAs can be incorporated into the nucleocapsids and then reverse transcribed into HBV DNA to generate defective HBV (dHBV) particles^9^,^10^,^11^,^12^. The level of dHBV particles in the sera of patients with chronic hepatitis B (CHB) was shown to be related with liver disease^13^,^14^ and was enhanced prior to development of HCC^15^. spHBV RNAs also serve as the translation templates for a number of non-canonical HBV proteins. HBV splice-generated protein (HBSP) is one of the major spHBV RNAs-encoded proteins which was firstly identified by Soussan *et al.* in the livers of CHB patients^16^, and has been reported to be associated with viral replication, liver diseases and cancer development^17^,^18^,^19^. In spite of these evidences suggesting that HBV splicing is a common event during HBV infection and may be involved in the pathogenicity or persistence of HBV, the clinical and biological significance of HBV splicing needs to be further defined.

Interferon-α (IFN-α), a cytokine with antiviral and immunomodulatory activities, has been approved for treatment of HBV infection since 1990s. However, IFN therapy is effective in only 30–40% of CHB patients^20^,^21^. One possible explanation for the failure to respond to IFN therapy in CHB patients is that HBV has developed strategies to counteract the IFN signaling transduction^22^,^23^,^24^,^25^,^26^,^27^. The Pol protein consisting of terminal protein (TP), reverse transcriptase (RT), RNaseH (RH), and a non-conserved spacer domain between the TP and RT domains has been shown to be multifunctional, not only playing essential role during viral replication but also having modulatory functions by interacting with a number of host factors^28^. Foster *et al.* found that the expression of the TP region of Pol inhibits cellular responses to IFN-α^22^,^23^, and we further observed that the TP and RH domains of Pol inhibit IFN-activated STAT1 serine 727 phosphorylation and STAT1 and STAT2 nuclear translocation, respectively^25^. Since Pol is believed to be produced at a relatively low level during viral replication, the real physiological relevance of these findings remains to be classified.

Considering that most of the spHBV variants contain sequences that could be translated into proteins which contain domains with anti-IFN activities derived from the open reading frame (ORF) of Pol gene^11^,^29^, we hypothesized that there may be a correlation between HBV spliced variants and IFN responses. To test this hypothesis, we collected serum samples from CHB patients before and after IFN therapy to analyze the relationship between the productions of HBV spliced variants and the failure to respond to IFN therapy. In addition, we examined the expression of HBV spliced variants and investigated the potential role of HBV splicing in counteraction of IFN signaling in cell culture systems and in a mouse model.

## Results

### The level of spHBV variants in the sera of CHB patients negatively correlates with IFN responsiveness

**Figure 1:**
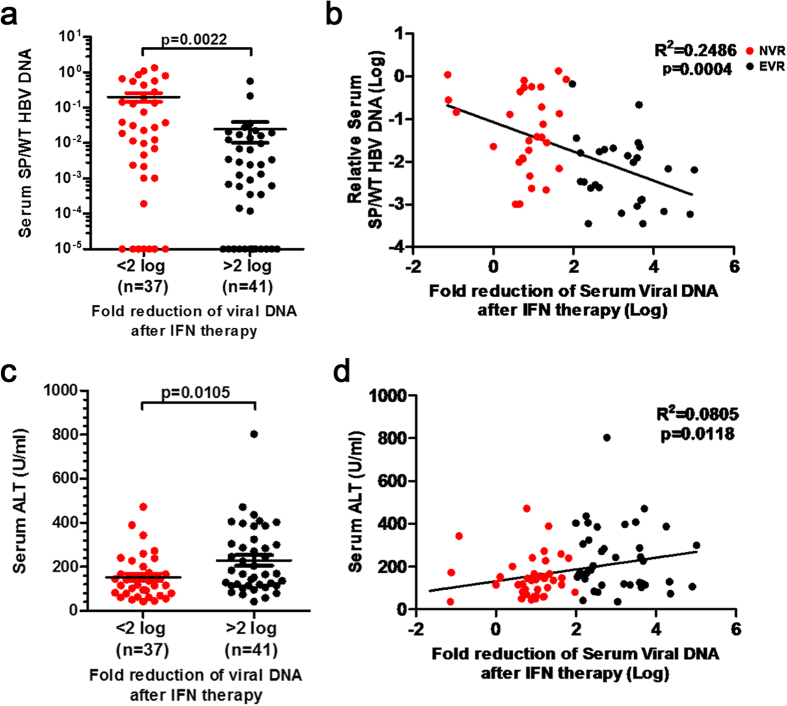
The level of spHBV variants correlates with IFN responsiveness. The ratio of spHBV to wtHBV DNAs (**a**) or the level of ALT (**c**) before IFN therapy in serum samples was compared between NVRs and EVRs using Student’s t-tests. The correlation between the fold reduction of serum viral DNA following 3 months of IFN therapy and the proportions of spHBV was analyzed in samples with spHBV/wtHBV > 0.0005 using Spearman’s correlation (**b**). The correlation between baseline serum ALT level and the fold reduction of serum viral DNA after IFN therapy was analyzed using Spearman’s correlation (**d**).

To reveal the relationship between spHBV variants and the responsiveness to IFN therapy, we collected 78 paired serum samples from CHB patients before and after IFN therapy, classified as either non-virological responders (NVRs, n = 37) or early-virological responders (EVRs n = 41) based on whether 2-log_10_ of viral load reduction was achieved 12 weeks after starting IFN therapy. The level of spHBV DNAs and wtHBV DNAs in the sera (before IFN therapy) was quantified by qPCR. The ratio of spHBV to wtHBV DNAs was nearly eight-fold higher in NVRs compared to EVRs (Fig. 1a). We further applied the multiple linear regression model to determine whether independent variables including spHBV/wtHBV (Log), gender, age, ALT and HBsAg (Log) are making a significant contribution to the fold of viral DNA reduction after IFN therapy (Table S3). We found that the ratio of spHBV/wtHBV negatively correlated with the fold of viral DNA reduction after 12 weeks of IFN therapy (Fig. 1b), implicating an association between the productions of spHBV variants and poor responses to IFN therapy. Consistent with previous reports^30^,^31^, a higher baseline ALT level was also found to correlate with a better outcome of IFN therapy (Fig. 1c,d). We did not observe a correlation between spHBV and ALT (Fig. S3B) or between spHBV and HBsAg (Fig. S3C) and there was no significant difference in the spHBV/wtHBV ratio between males and females (Fig. S3D).

### Identification of the types and frequencies of distinct spHBV variants in the sera of patients

**Figure 2:**
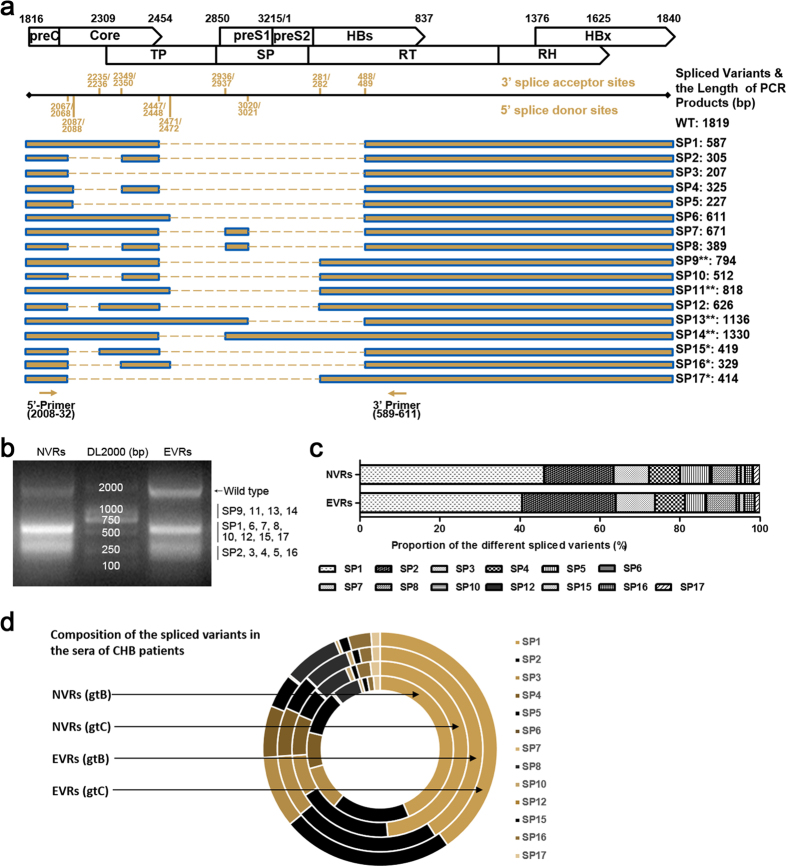
Species of the spHBV variants identified in the sera of CHB patients before IFN therapy. Schematic representation of the ORFs of HBV genome, the known accept and donor sites for HBV splicing, the reported spHBV variants (**undetected in the current 454 sequencing), the newly identified spHBV variants (*), and the primers designed for PCR amplification of the spHBV variants (**a**). The HBV DNAs extracted from sera were PCR amplified, and the amplicons were then separately grouped into NVRs and EVRs and pooled, and then subjected to agarose gel electrophoresis (**b**). The proportions of each of the spHBV variant in NVRs and EVRs according to the 454 sequencing data (**c**). The composition of spHBV variants in NVRs and EVRs with HBV genotype B and C according to the 454 sequencing data (**d**).

To date, a series of splice donor and acceptor sites in the HBV genome and at least fourteen species of spHBV variants have been identified^11^,^15^,^29^ (Fig. 2a). We speculated whether there was any difference in the distributions of spHBV types between CHB patients who had better or poorer responses to IFN therapy. Therefore, we amplified spHBV DNAs from the sera of the 78 patients obtained before IFN therapy. The PCR products from NVRs and EVRs (Fig. 2b) with genotype B or genotype C were separately pooled for high-throughput sequencing (Fig. S4). Through the 454 GS Junior sequencing followed by BLAST analysis, we identified ten reported spHBV variants in both of the two groups. Besides, we identified three species of spHBV DNAs that have not been reported previously named as SP15, SP16 and SP17, though all of the splice acceptor and donor sites identified in this study have been reported previously. However, SP9, SP11, SP13 and SP14 were not detected, which may be due to the limitation of the 454 sequence read length. Analysis of the frequencies of each species of the spHBV showed that SP1, SP2 and SP3 accounted for over 70% of the total spHBV variants in both NVRs and EVRs group (Fig. 2c and Table S1). Interestingly, we found that while the distribution of the species of spHBV variants was similar between EVRs with HBV genotype B and with HBV genotype C, the proportion of the major spliced variant SP1 in NVRs with HBV genotype C (48.6%) was higher than that with HBV genotype B (43.6%) (Fig. 2d and Table S1), which together with the previous reports^32^,^33^ suggested that the production of spliced variants during HBV infection is somehow genotype-specific.

### The spliced HBV DNAs detected in the sera may reflect the intracellular status of HBV RNA splicing

**Figure 3:**
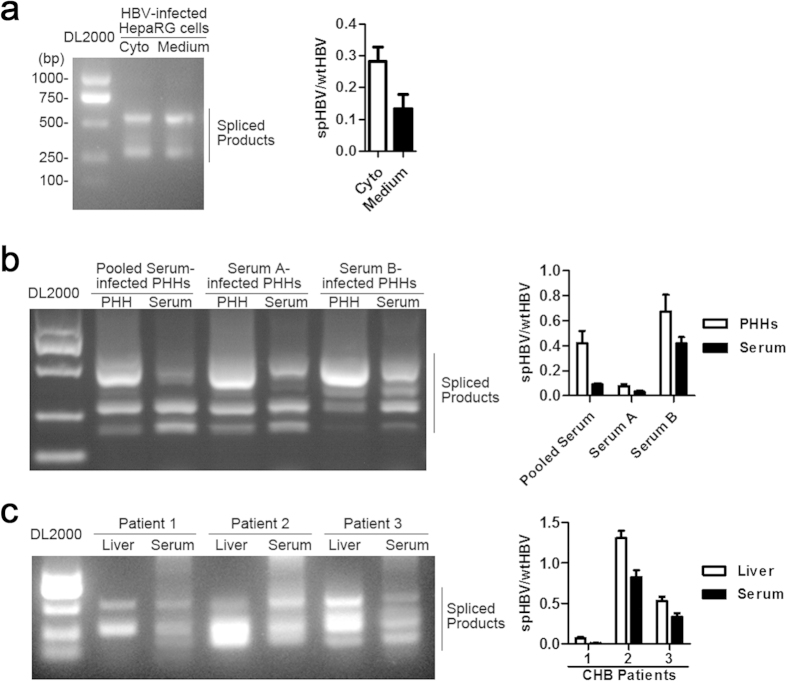
Detection of the intra- and extra-cellular HBV spliced variants. The cDNAs reverse-transcripted from the total RNAs extracted from HBV-infected HepaRG cells (**a**), PHHs (**b**), or the livers from CHB patients (**c**) and the HBV DNAs extracted from the cell culture medium (**a**), the sera used for PHHs infection (**b**), or the paired sera (**c**) were analyzed by PCR followed by agarose gel electrophoresis (the left panel) and real-time PCR (the right panel).

It has been shown that the HBV spliced RNAs can be packaged into the capsids and reverse transcribed into HBV DNA, leading to the secretion of dHBV particles^9^,^10^,^11^,^12^,^13^. To investigate whether the spHBV DNAs detected in the sera could reflect the viral spliced products distributed in HBV-replicating cells, we studied the expression of intra- and extra-cellular spHBV variants in HBV-infected cell culture systems and in paired sera and liver samples from CHB patients. In HBV-infected HepaRG cells, the intracellular spHBV RNAs had the similar splicing pattern as the spHBV DNAs extracted from the cell culture medium. However, the ratio of spHBV/wtHBV RNAs in HBV-replicating cells were about two-fold higher than the ratio of spHBV/wtHBV DNAs in dHBV particles in the culture medium (Fig. 3a). In HBV-infected primary human hepatocytes (PHHs), the intracellular spHBV RNAs and the spHBV DNAs extracted from the serum used for HBV infection of PHHs showed similar PCR pattern (Fig. 3b). The comparison of the patterns of HBV spliced products detected in paired liver and sera samples from three CHB patients also revealed close relationships between the expression of spHBV variants in livers and in sera, and the proportions of spHBV variants was higher in livers than that in sera (Fig. 3c), which was consistent with the results in cell models. These data suggested that the spHBV DNAs detected in the sera could, to some extent, reflect the states of viral RNAs splicing in HBV-infected cells.

### The translational products of spHBV RNAs inhibit IFN signaling transduction

**Figure 4:**
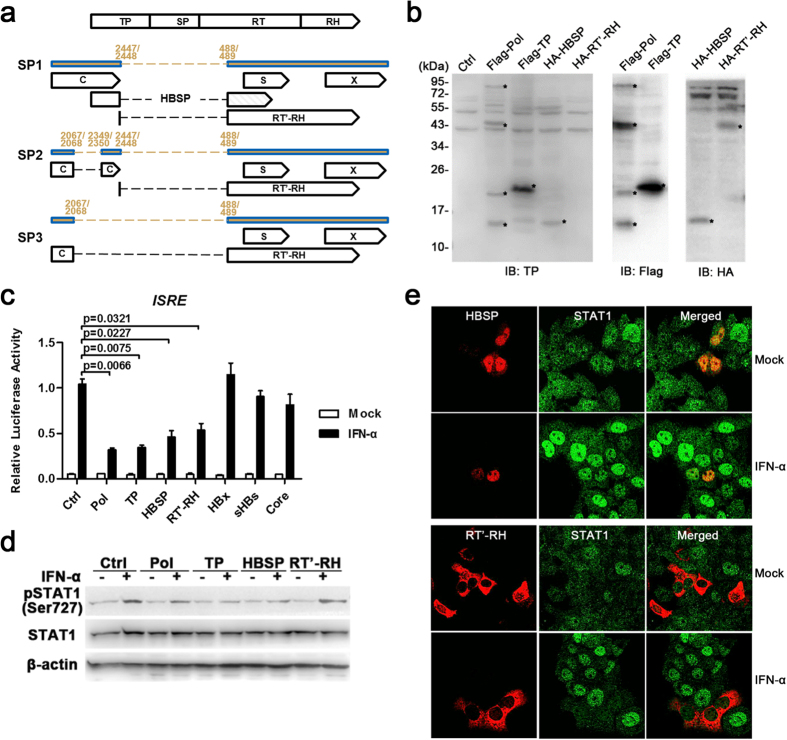
Spliced HBV RNAs-encoded HBSP and RT’-RH inhibit IFN signaling. Schematic representation of the putative ORFs present in SP1, SP2 and SP3 (**a**). The expression of proteins in Huh7 cells transfected with indicated plasmids were analyzed by immunoblotting with indicated antibodies (**b**). Huh7 cells transfected with the indicated plasmids were mock- or IFN-α–treated for 12 h and then harvested for luciferase reporter assay. The data was analyzed using Student’s t-tests (**c**). Huh7 cells transfected with the indicated plasmids were treated with IFN-α or mock treated for 1 hour and then harvested for immunoblotting (**d**), or for the immunofluorescence assay (**e**).

It has been shown that spHBV RNAs contain encoding sequences from which a number of non-canonical viral proteins, including truncated HBc, N-terminal-truncated Pol (RT’-RH), truncated small HBs, and HBSP, could be translated^11^,^29^, however, the biological functions of these proteins needs to be further defined. Considering that the level of spHBV variants was significantly increased in patients who had poorer responses to IFN therapy, we speculated whether the splice-generated translational products expressed in HBV-replicating cells had anti-IFN effects. IFN-α regulates hepatic genes expression and initiates antiviral responses through activation of the evolutionary conserved JAK-STAT pathway^27^,^34^. Since we have demonstrated that the Pol protein is an active antagonist of IFN responses that inhibits IFN-α signaling transduction via its TP and RH domains^25^,^26^, we primarily focused on HBSP and N-terminal-truncated Pol (RT’-RH), of which the former contains truncated TP domain and is the most well-known protein products of HBV splicing while the latter contains RH domain and is common in most of the known spHBV variants (Fig. 4a). The expression of HA-tagged-HBSP and -RT’-RH were verified by western blot analysis using antibodies against TP and HA (Fig. 4b). Reporter assay showed that HBSP and RT’-RH exhibited strong inhibition of IFN-induced ISRE activation that was comparable to Pol and TP, while HBx, sHBs and HBc did not show any inhibitory effect (Fig. 4c). Furthermore, HBSP and RT’-RH were shown to suppress IFN-induced STAT1 Ser-727 phosphorylation and STAT1 nuclear translocation, respectively (Fig. 4d,e). Impaired IFN-activated STAT1 and STAT2 nuclear translocation was also observed in HBV-infected HepaRG and PHH cells (Fig. S8).

**Figure 5:**
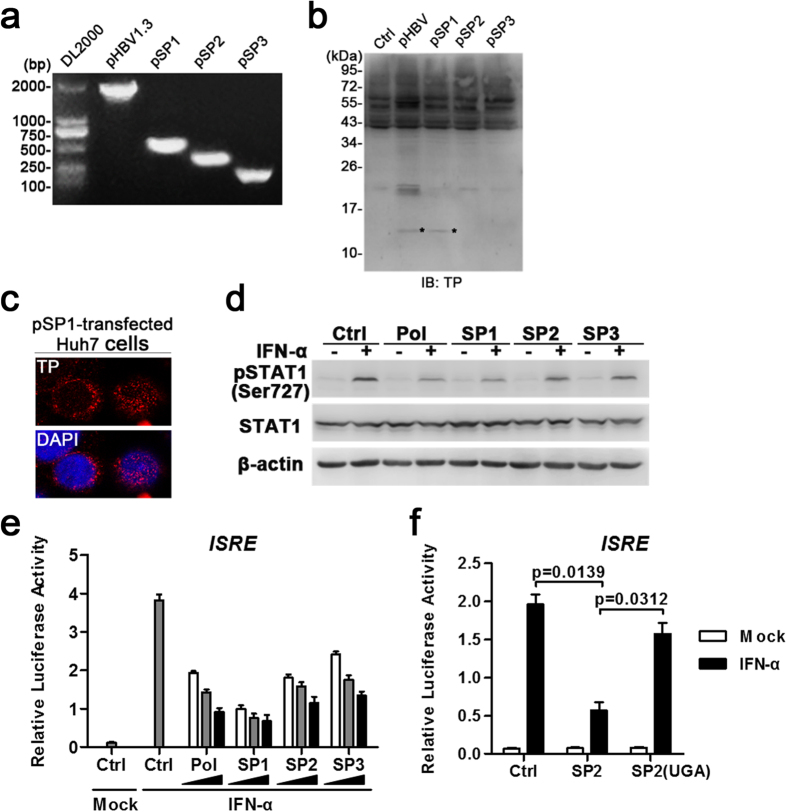
The effect of HBV spliced variants 1, 2 and 3 on IFN signaling. The cDNAs reverse-transcripted from the total RNAs extracted from the cells transfected with indicated plasmids were analyzed by PCR with the SP primer followed by agarose gel electrophoresis (**a**). The expression of proteins (*most likely to be the HBSP) in Huh7 cells transfected with indicated plasmids were analyzed by immunoblotting with anti-TP (**b**). Immunofluorescence assay was performed to detect the expression and cellular distribution of proteins containing TP domain in pSP1-transfected Huh7 cells using anti-TP Abs (**c**). Huh7 cells transfected with the indicated plasmids were mock- or IFN-α–treated for 1 hour and then harvested for immunoblotting (**d**), or for 12 h and then harvested for luciferase reporter assay (**e**,**f**). The data was analyzed using Student’s t-tests.

Since SP1, SP2 and SP3 were identified as the dominant species of spHBV variants in sera, we further cloned SP1-3 sequences from the cDNAs of pHBV-transfected cells (Fig. 5a). The expression of proteins in cells transfected with SP1 were detected using western blot and immunofluorescence with anti-TP antibodies. A protein around 15kD, that most likely to be HBSP, was detected in pHBV-, pSP1- but not pSP2- and pSP3-transfected cells (Fig. 5b). The proteins detected in pSP1-transfected cells using TP antibodies distributed in both of the cytoplasm and nucleus (Fig. 5c). While IFN-activated STAT1 Ser-727 phosphorylation was only suppressed in pSP1-transfected cells (Fig. 5d), ISRE activation was suppressed in all the three groups that transfected with pSP1, pSP2 and pSP3 (Fig. 5e). Moreover, introduction of a stop codon into the RT’-RH ORF in pSP2 (Fig. 5f) partially restored SP2-mediated suppression of ISRE activation, indicating that the inhibitory activities of spHBV depend on the generation of spHBV RNAs-encoded proteins.

### HBV splicing plays a role in viral suppression of IFN responses

**Figure 6:**
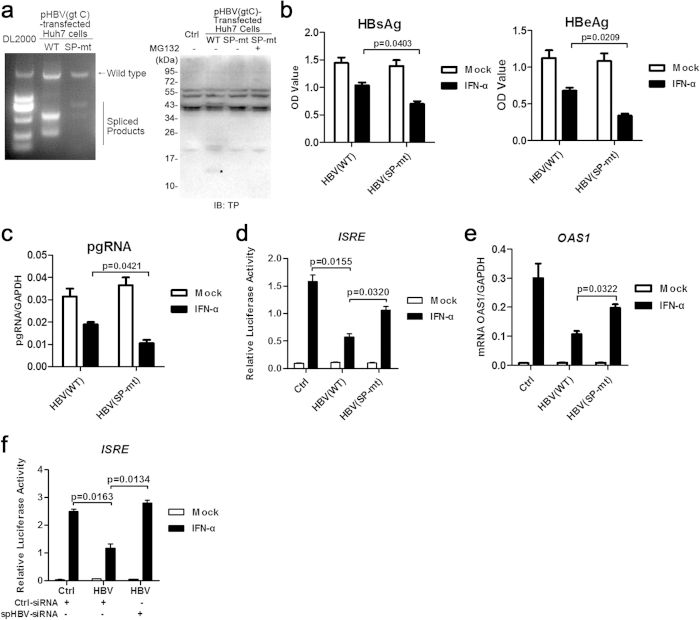
Mutation of the common splice sites of HBV impaired its anti-IFN activities in Huh7 cells. The cDNAs reverse-transcripted from the total RNAs extracted from pHBV1.3- or pHBV1.3-SP-mt-transfected Huh7 cells were analyzed by PCR followed by agarose gel electrophoresis (**a**, the left panel). The expression of proteins in Huh7 cells transfected with indicated plasmids was analyzed by immunoblotting using anti-TP antibodies (**a**, the right panel). The level of HBsAg and HBeAg in culture supernatants 48 h post transfection was analyzed by ELISA (**b**). Huh7 cells were transfected with pHBV or pHBV(SP-mt) plasmids. IFN-α was added to the culture medium 24 h post transfection. Following an additional 24 h of culture, the RNAs were extracted for real-time PCR (**c**). Huh7 cells transfected with the indicated plasmids were mock- or IFN-α–treated for 12 h and then harvested for luciferase reporter assay (**d**), or for 6 h and then RNAs were extracted for real-time PCR (**e**). Huh7 cells co-transfected with pHBV and control siRNAs or siRNAs targeting spHBV RNAs were mock- or IFN-α–treated for 12 h and then harvested for luciferase reporter assay (**f**). The data was analyzed using Student’s t-tests.

To further examine the role of HBV splicing in viral inhibition of IFN responses in the context of viral replication, pHBV1.3-SP-mt was constructed by introducing two mutations of the common spliced acceptor and donor sites of HBV genome into pHBV1.3. Mutation of the two sites did not change the amino acids of either the Pol or the HBs proteins but, as expected, resulted in an impaired generation of the spliced RNAs and the protein products containing TP domain (Fig. 6a). There was no significant difference in the expression level of HBsAg, HBeAg and pgRNA between cells transfected with pHBV-wt and pHBV-SP-mt. However, pHBV-SP-mt appeared to be more sensitive in response to IFN treatment compared with pHBV-wt, as evidenced by greater suppression of viral antigens and pgRNA production upon IFN treatment (Fig. 6b,c). Simultaneously, there was a higher ISRE-driven luciferase expression (Fig. 6d) and OAS1 mRNA transcription (Fig. 6e) upon IFN treatment in the pHBV-SP-mt group compared with the pHBV-wt group. Similar results were also obtained in HepG2 cells (Fig. S9). In addition, we designed siRNA targeting the major spHBV RNAs (Fig. S10) to further validate the role of HBV spliced products in anti-IFN effects. The results showed that the down-regulation of spHBV RNAs by using the siRNA resulted in a significant restoration of the IFN-induced ISRE activation (Fig. 6f).

**Figure 7:**
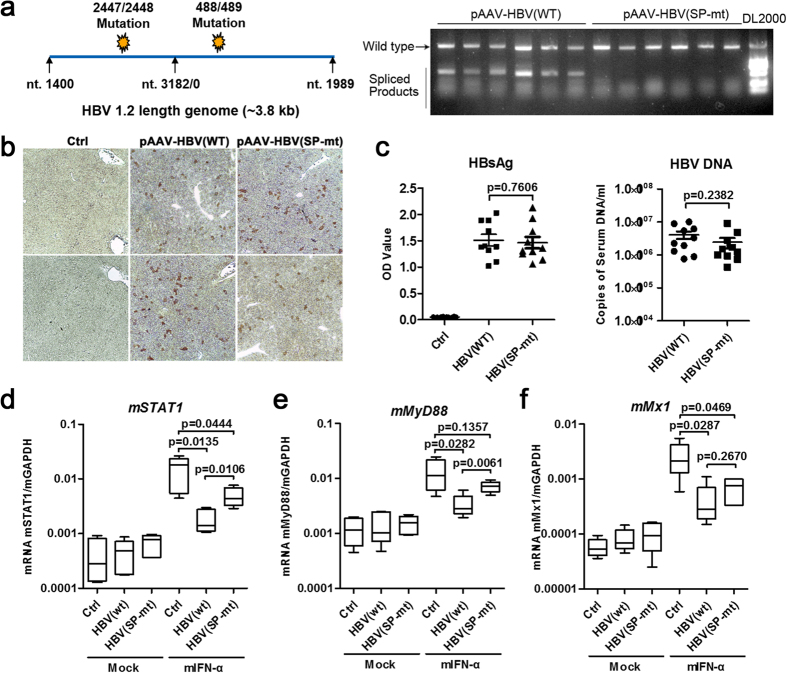
Mutagenesis analysis of the role of HBV splicing in viral anti-IFN effects in the hydrodynamic injection-based mouse model. pAAV/HBV1.2(SP-mt) was constructed by introducing two mutations of the common spliced acceptor and donor sites of HBV genome into pAAV/HBV1.2 and the mutations resulted in an impaired generation of spliced RNAs (**a**). Immunohistochemical staining for HBcAg in hepatocytes of mice injected with control vectors or the pHBV(wt) and pHBV(SP-mt) plasmids (**b**). Serum levels of HBsAg and viral DNA of the mice injected with indicated plasmids (2 weeks post injection) were determined by ELISA and qPCR, respectively (**c**). Intrahepatic RNA levels of mSTAT1 (**d**), mMyD88 (**e**), and mMx1 (**f**) were analyzed 6 h after mIFN-α or saline treatment in 6 different experimental groups (n = 5 in each group). The data was analyzed by one-way analysis of variance followed by Bonferroni tests.

To confirm the effect of HBV splicing on IFN responses *in vivo*, a replication-competent HBV vector with two common spHBV sites mutation was constructed and used for mouse hydrodynamic injection (Fig. 7a). The mutation did not result in a significant change in the production of viral antigens and DNAs in the liver in the group injected with mutated plasmid compared with the wild-type group (Fig. 7b,c). However, the mIFN-α-mediated induction of mSTAT1 and mMyD88 was significantly increased in the livers of pHBV(SP-mt)-injected mice compared with that of pHBV(wt)-injected mice (Fig. 7d,e). Taken together, these results suggested that HBV splicing may contribute to the viral counteraction of the IFN responses.

## Discussion

Like many other viruses that produce spliced viral RNAs via utilization of the human splicing mechanism^35^, HBV encodes specific sequence elements to promote splicing of viral RNAs^29^. Although there have been several reports about the association between spHBV variants and viral replication and pathogenesis, the biological and clinical significance of HBV splicing remains obscured. Here, we found a correlation between the productions of spHBV variants and the impaired responses to IFN therapy. We identified the species of spHBV variants in sera of HBV-infected patients, and showed that the major translational products of the spHBV RNAs had anti-IFN activities in hepatic cells while the mutations of the splicing-related sites attenuated the viral anti-IFN activities. Together, these data revealed an association between the productions of spHBV variants in the sera and impaired responses to IFN therapy, and thus provided a novel molecular mechanism by which HBV resists the IFN treatment (Fig. S11).

According to the previous studies^7^,^15^, we used the ratio of spHBV/wtHBV rather than the value of absolutely amounts of spHBV products to evaluate the level of HBV splicing. Notably, the ratio of spHBV/wtHBV varied from 0.00001 to 1.351 (the median is 0.0167) in sera collected from CHB patients, demonstrating that spHBV variants represented a relatively minor population in the sera of majority of the patients in comparison with the wtHBV genomes. However, the proportion of intracellular spHBV variants in HBV-transfected or -infected cells (0.10–0.60) was significant higher than that in the sera. The difference between the intra- and extra-cellular spHBV/wtHBV ratio may be explained by the processing of dHBV particles containing spliced genomes may have lower efficacy compared to that of wtHBV secretion^36^. In spite of this, the patterns of intra- and extra-cellular spHBV variants were similar (Fig. 3), suggesting that the frequencies of the multiple spHBV variants identified in sera could partially reflect the status in the livers.

IFN-α has been used to treat CHB but is effective in a small portion of patients. Several clinical, biochemical and virological factors, including female sex, higher baseline alanine aminotransferase (ALT) levels, low baseline HBV DNA and on-treatment HBsAg dynamic, have been shown to be the prognostic parameters for sustained response following the IFN therapy^30^,^31^,^37^. Here, the proportion of spHBV variants was shown to be associated with the responses to IFN therapy, but did not associate with gender, ALT, viral load or HBsAg (Fig. S3), and was not much affected by IFN treatment (Fig. S6), suggesting that it is a relatively independent factor contributing to the prognosis of IFN therapy and may represent an adjunct to guide therapeutic decision making before and during IFN therapy. We have demonstrated that the major protein products generated from the spHBV RNAs had anti-IFN responses, thus providing a molecular explanation for this association between the spHBV variants and the IFN responses. However, the mechanisms involved in regulating the efficacy of HBV splicing remain unclear.

Previous studies suggested that the production of dHBV particles is dependent on the liver status^14^ and the expression of HBSP is associated with viral replication and liver fibrosis^17^. Moreover, different HBV genotypes were reported to display diverse splicing patterns and levels^32^,^33^. Here, the variation in the ratio of spHBV/wtHBV among different HBV genotypes was observed in the HBV-transfected cell models in a A < B < C < D manner (Fig. S5B), which may be associated with the clinical observation suggesting that the response rate to IFN among CHB patients can be correlated with the genotypes as A > B > C > D^31^,^38^. The regulatory mechanism by which different HBV genotypes generate diverse splicing patterns is still unclear. Huang *et al.* suggested that different HBV genotypes exhibit distinct preferences in the usage of splice donor/acceptor sites and thus generate diverse splicing patterns^32^, though the splicing-related sites among different HBV genotypes are conserved (Fig. S5A). Nevertheless, it is necessary to further confirm and investigate the HBV genotype-specific splicing patterns and levels in the sera and livers of CHB patients in some larger cohorts and to further clarify the related molecular mechanisms.

Although the regulatory mechanisms involved in protein translation from the HBV pgRNAs and spliced RNAs remain to be defined, several non-canonical HBV proteins generated by the HBV spliced RNAs including HBSP, HBDSP, Pol’-HBs’ fusion proteins have been identified and were reported to be associated with viral replication, cell apoptosis, and severity of liver disease^17^,^18^,^19^,^29^,^36^,^39^. Here we found that ectopic expression of the major translational products of spHBV including HBSP and RT’-RH exhibited strong inhibition of IFN signaling, while mutation of the common splicing sites in HBV DNA attenuated the inhibitory effect of HBV on IFN-activated responses. In addition, previous reports have shown that spHBV variants in the hepatic cells lead to the intracellular accumulation of HBc proteins, which are able to inhibit IFN-mediated MxA induction^10^,^40^. Together, these data illuminate a novel mechanism where HBV splicing provides a source of proteins with anti-IFN activities which contribute to the viral suppression of the IFN responses. Thus, development of an approach that aims at reducing the level of viral spliced variants may be advantageous to improve the efficacy of IFN-α therapy as well as to reduce the risk of HBV-induced liver diseases.

HBV Pol has been demonstrated to play a key role in HBV-mediated inhibition of IFN responses^22^,^23^,^25^,^26^,^41^,^42^. CHB patients whose liver specimens contained large numbers of cells expressing TP tend to poorly respond to IFN treatment^23^, and TP and RH domains exhibit a similar inhibitory effect on IFN signaling compared to the full-length Pol in transfection cell models^25^. Notably, the full-length Pol is believed to be produced at a limited level during viral replication^43^. In contrast, spHBV RNAs that serve as templates for translation of splice-originated viral proteins are produced in abundant amounts^3^,^4^,^5^,^7^. Interestingly, most of the reported and putative splice-generated proteins contain domains with anti-IFN activities derived from the ORF of Pol gene. Using a rabbit polyclonal antibodies against TP, a protein around 15kD, that most likely to be HBSP, was detected in pFlag-Pol-, pHBV-, and pSP1-transfected cells (Figs 4b and 5b), and nuclear and cytoplasm staining were observed in hepatocytes from liver biopsies of CHB patients (Fig. S2C). Due to the lack of antibodies against the RT or RH domains of Pol, we were not able to demonstrate the RT’-RH expression at present, but the introduction of a stop codon into the ORF encoding RT’-RH located in SP2 abolished the inhibitory effects of SP2 on IFN signaling, implicating that the inhibitory effects of SP2 depend on the RT’-RH protein expression. This is similar to a recent report suggested that enhancing the replicative effect of a 2.2 kb double-spliced HBV variant required an intact HBV X expression cassette^44^. Moreover, the expression of viral proteins in cells transfected with pFlag-Pol and HBV-replicating or -infected cells was examined using antibodies against TP and SP, and the results showed that the ectopic expression of Flag-Pol could be detected by both of the anti-TP and anti-SP antibodies, while protein expression in the HBV-replicating cells could only be detected by anti-TP antibodies but not anti-SP antibodies (Fig. S7). These data support the idea that the expression level of full-length Pol is relatively low in the context of viral replication while proteins originated from HBV splicing which contain Pol ORF-derived domain(s) that have anti-IFN activities are largely expressed. More studies are required to further examine the expression and function of the splice-generated viral proteins in liver tissues.

## Materials and Methods

### Patients and Clinical Specimens

Seventy-eight paired serum samples before and after treatment were obtained from CHB patients who underwent PEG-IFN or conventional IFN treatment^45^ in Shanghai Public Health Clinical Center and Huashan Hospital affiliated to Fudan University. All 78 patients had not received nucleoside analogues or IFN therapy within 6 months of initiation of the study. Most of the patients enrolled were HBeAg positive (70/78). The clinical characteristics of the enrolled patients were presented in Table S2 in detail and summarized in Table 1. Exclusion criteria included co-infection with HCV or HIV and decompensated cirrhosis.

### Plasmids

pHBV1.3-AF411411 (genotype C) was a gift from Dr. Yongxiang Wang and Prof. Yumei Wen of Fudan University^44^. pcDNA3.1-3 × Flag-Pol/-Core/-sHBs/-HBx/-TP was described previously^41^. pAAV/HBV1.2 was a kind gift from Prof. Pei-jer Chen of National Taiwan University. The pISRE-Luc and the pRL-TK were purchased from Stratagene and Promega, respectively. pcDNA3.1-2×HA-HBSP and p cDNA3.1-2×HA-RT’-RH were constructed as follows: The coding sequences encoding HBSP (2309–2449+491–709) and RT’-RH (2448–2449+491–1625) were amplified from cDNA of pHBV1.3-AF411411-transfected Huh7 cells and inserted into pcDNA3.1-2×HA vector. pSP1, pSP2 and pSP3 were constructed as follows: the DNA sequences amplified from the cDNA templates which were obtained by reverse transcription of total RNAs extracted from pHBV1.3-transfected Huh7 cells with primers corresponding to amino acids 1903 to 1934 (forward) and 1595 to 1625 (reverse) of the HBV genome AF411411 were inserted into the multiple cloning sites (MCS) of the pGEM-T Easy Vector (Promega) followed by a blue-white screening step, positive clones containing SP1, SP2 and SP3 sequences were identified by sequencing and then subcloned into the pcDNA3.1 mammalian expression vector. pSP2(UGA) is a mutant of pSP2 with the GGA at 585–587 corresponding to the HBV genome AF411411 changed to TGA to make it deficient for truncated-polymerase synthesis without altering HBs. pHBV1.3(SP-mt) and pAAV/HBV1.2(SP-mt) are mutants of pHBV1.3 and pAAV/HBV1.2, respectively, with the CCAG/GA (488/489) changed to CGTCCA and AAT/GTT (2447/2448) changed to ATCCTT.

### Antibodies and Regents

The antibodies used in this study included the following: anti-β-actin and anti-Flag (mouse, Sigma, China), peroxidaseconjugated secondary goat anti-mouse and anti-rabbit Abs (Santa Cruz, China), anti-HBs (mouse, ZhongshanJinqiao), anti-HBc (rabbit, ZhongshanJinqiao), anti-HA (mouse, Cell signaling, China), anti-STAT1 and anti-STAT2 (rabbit, Cell Signaling, China), and Alexa 488- or Cy3- coupled secondary Abs (Jackson). Polyclonal antibodies against HBV polymerase TP or Spacer were prepared by immunizing rabbits with GST-TP or GST-Spacer fusion proteins respectively through cooperation with ABclonal Biotech of China (Fig. S2). Briefly, the DNA sequences that encode the terminal protein or spacer region of HBV polymerase were PCR amplified from pHBV1.3-AF411411. The restricted digested PCR products were cloned into pGEX4T-1. The constructs encoded fusion proteins consisting of GST at the N terminus and TP or SP at the C terminus. The recombinant proteins expressed in E. coli strain BL21 were purified using glutathione sepharose beads and then assessed by SDS-PAGE. New Zealand White rabbits were injected subcutaneously with 600 μg of purified GST-TP or GST-SP mixed with an equal volume of complete Freund’s adjuvant. The immunization was repeated at 3 and 5, and 7 weeks with 300 μg of antigens every time after the first injection. The animals were bled 1 week after the fourth injection and then once monthly. Antibody titers were determined by a neutralization assay using ELISA. Polyclonal antibodies obtained from the serum of immunized rabbits were purified by affinity chromatography and tested for reactivity to the fusion proteins by immunoblot analysis before application. The siRNAs targeting the major spliced HBV RNAs (Fig. S10) were purchased from Ribobio (China). Human and mouse recombinant IFN-α were purchased from Calbiochem and PBL respectively. A dose of 500 U/ml of IFN-α was used in the cell culture systems. The mice were intraperitoneally injected with 500 U/g body weight mIFN-α.

### Cells and HBV Infection

The human hepatoma cell lines Huh7 and HepG2 obtained from the Cell Bank of the Chinese Academy of Sciences were routinely cultured as described^25^. Differentiated HepaRG cells and primary human hepatocytes (PHHs) were purchased from BIOPREDIC INTERNATIONAL (France) and Shanghai RILD Inc. respectively. To obtain the differentiated HepaRG (dHepaRG), the cells were cultured for 2 weeks in standard medium, then for 2 more weeks in medium supplemented with 1.8% dimethylsulfoxide according to the manufacturers’ instructions. Primary human hepatocytes were cultured in Williams E medium supplemented with 5% FBS, 5 μg/ml transferrin, 5 ng/ml sodium selenite, 3 μg/ml insulin, 2 mM L-glutamine, 100 U/ml penicillin and 100 μg/ml streptomycin. HBV infection of the hepatocytes was performed as described previously^42^. The dHepaRG cells or PHHs plated in the chamber (Lab-Tek, Thermo, China) were incubated overnight with HBV-positive sera (1 volume of infectious pooled serum from 10 CHB patients diluted in 10 volumes of culture medium containing 5% PEG 8000). After incubation, cells were rinsed three times. The HBeAg secretion and the viral DNA in the medium were determined every 3 days post infection (data not shown). The cells successfully infected with HBV were used for further treatment or assay 10 days post-infection.

### Animal studies

The hydrodynamic injection of HBV DNA into the tail vein of C57BL/6 mice was performed as previously described^25^.

### DNA and RNA extraction, RT-PCR and Real-time PCR

The HBV DNAs in the sera were extracted by the sodium octanoate method. Briefly, 2 volumes of serum was mixed with 1 volume of sodium octanoate solution (120 mM), boiled for 10 minutes and centrifuged at 13000 g for 10 minutes, the supernatant was collected for PCR assay. Total RNAs from cells were extracted using the TRIzol reagent (TianGen, China) and reverse-transcribed to cDNAs using ReverTra Ace®qPCR RT Kit (Toyobo, China) according to the manufacturer’s protocol. The DNA and cDNA samples were used as templates for PCR or real-time PCR. The PCR was performed by using the Taq PCR mix (Laifeng, Shanghai) with primers, as described in Fig. 2a, using the following cycle: 2 min at 94 °C for initial denaturing, followed by 36 cycles of 94 °C for 15 s, 58 °C for 30 s, and 72 °C for 40 s, the products were then analyzed by agarose gel electrophoresis. The real-time PCR for quantitation of wtHBV and spHBV were performed by using specific primers^15^,^46^ and thermo-cycling parameters described in Fig. S1. Each qPCR was carried out in duplicate using the SYBR Green Master Mix (Roche) in a StepOne Real-time PCR system (ABI, 96 or 384 well). The results are representative of three independent experiments.

### High-throughput 454 GS Junior Sequencing and Analysis

Four pooled PCR amplified products (NVRs-gtB, NVRs-gtC, EVRs-gtB, EVRs-gtC) containing ~200–800 bp DNA fragments were prepared as shown in Fig. S4. The amplicons were purified with the microconcolumns (Qiagen) before sequencing, the products of which were used to generate multiplex identifier (MID)-tagged libraries for 454 sequencing (Roche) according to the instructions of the manufacturer. Sequence reads from the 454/Roche GS Junior sequencer were subjected for performing BLAST with the reference sequences of HBV (2811–611) for identifying the spliced accept and donor site(s) of each read, according to which the reads were then classified into a number of species of spHBV variants. Only the species of spHBV that had a proportion of at least 0.01% among the total reads were calculated for analyzing the distribution of the spHBV variants.

### Immunofluorescence, Immunoblotting and Reporter assay

These assay were performed as previously described^25^.

### Statistical Analysis

Statistical analyses were performed by Student’s t-tests for comparison between two groups, the Spearman’s correlation analysis for association between spHBV and fold reduction of viral DNAs after IFN therapy, and one-way ANOVA followed by Bonferroni tests for multiple comparisons, using GraphPad Prism software. A value of p < 0.05 was considered statistically significant.

### Ethics Statement

Written, informed consent was obtained from all subjects prior to participation, and this study was approved by the ethics committee of Shanghai Public Health Clinical Center of Fudan University. The mouse experiments were conducted in accordance with the guide for the care and use of medical laboratory animals (Ministry of Health, China) and were approved by the ethics committee of School of Basic Medical Sciences of Fudan University.

## Additional Information

**How to cite this article**: Chen, J. *et al.* Hepatitis B virus spliced variants are associated with an impaired response to interferon therapy. *Sci. Rep.* **5**, 16459; doi: 10.1038/srep16459 (2015).

## Supplementary Material

Supplementary Materials

## Figures and Tables

**Table 1:** Summarized characteristics of the CHB patients.

	CHB Patients (n = 78)
Age (Range)	31 (18 ~ 55), Median (Range)
Male/Female	47/31
ALT(IU/ml)	192 (42 ~ 803), Median (Range)
HBsAg (U/ml)	6201 (250 ~ 72281), Median (Range)
HBeAg +/−	70/8
HBV DNA (Log Copies/ml)	7.48 (3.8 ~ >8), Median (Range)
HBV genotype (B/C/D)	21/55/2
