# Supplementary material for: Hepatitis B virus spliced variants are associated with an impaired response to interferon therapy
Source: Sci Rep. 2015 Nov 20;5:16459. doi: 10.1038/srep16459 (PMC4653653; doi:10.1038/srep16459)
Supplement: Supplementary Materials [file srep16459-s1.pdf]

Supplementary Materials

Hepatitis B virus spliced variants are associated with an impaired response to interferon therapy

Jieliang Chen[1], Min Wu[3], Fan Wang[3], Wen Zhang[1], Wei Wang[3], Xiaonan Zhang[3], Jiming Zhang[4], Yinghui Liu[1], Yi Liu[3], Yanling Feng[3], Ye Zheng[3], Yunwen Hu[3], Zhenghong Yuan[1.2]

1. *Key Laboratory of Medical Molecular Virology, School of Basic Medical Sciences, Shanghai Medical College of Fudan University, China*
2. *Institutes of Medical Microbiology and Biomedical Sciences, Fudan University, Shanghai, China*
3. *Shanghai Public Health Clinical Center, Shanghai Medical College of Fudan University, China*
4. *Huashan Hospital affiliated to Fudan University, China*

Correspondence
Prof. Zhenghong Yuan, MD, PhD
E-mail: zhyuan@shaphc.org
Add: Rm 1115, Zhidao Bldg, 138 YiXueYuan Rd, Shanghai 200032, China
Tel: +86-21-54237669

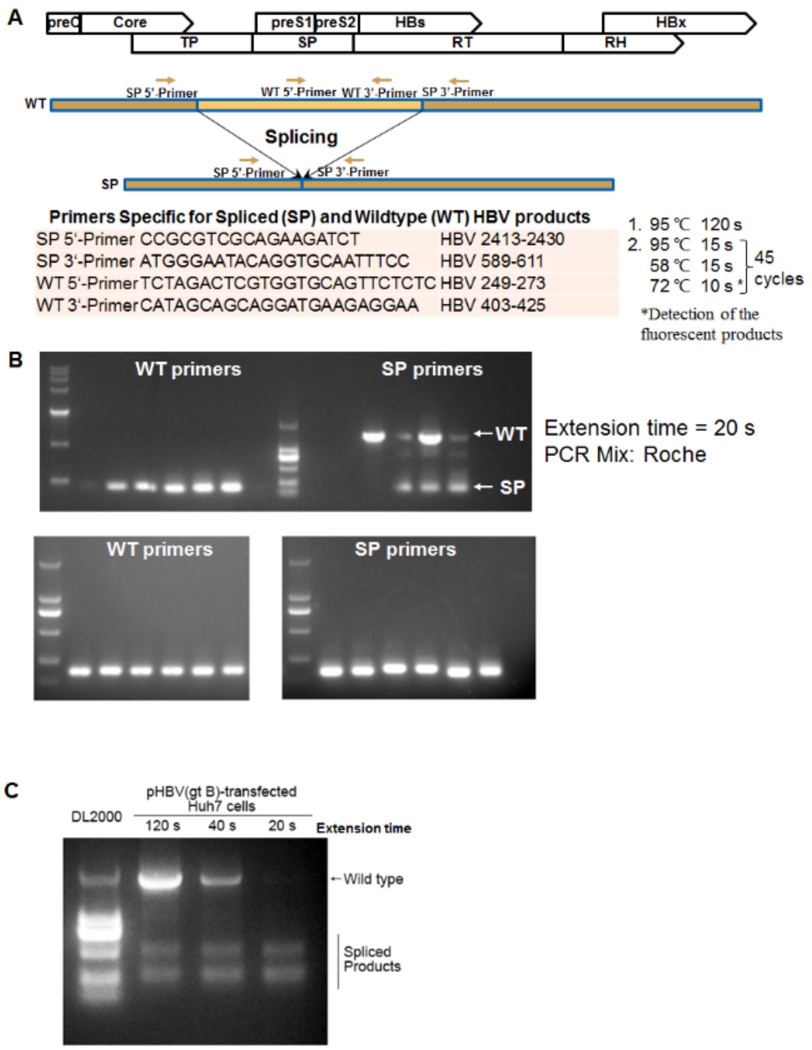

Fig S1. Optimization of the PCR protocol for WT and SP HBV DNAs amplification. (A) Schematic of the designed primers for detecting WT- and SP-HBV DNAs. (B) The HBV DNAs extracted from the sera of CHB patients were analyzed by PCR followed by agarose gel electrophoresis with primers specific for WT or SP. The extension time during PCR was 20 s (the upper panel) or 10 s (the lower panel). The data showed that protocol setting extension time as 10 s was suitable for amplify specific WT- and SP- HBV DNAs, during which there was no nonspecific amplification of WT products with SP primers. (C) The cDNAs reverse-transcripted from the total RNAs extracted from Huh7 cells transfected with pHBV1.3 were analyzed by PCR followed by agarose gel electrophoresis. The data showed that the shorter extension time leaded to less amplified products of wtHBV (~2 kb) but did not much affect the amounts of the amplified products of spHBV(~0.3-0.5 kb), which may be a result of the characteristic of the polymerase (1 kb/min) used in PCR.

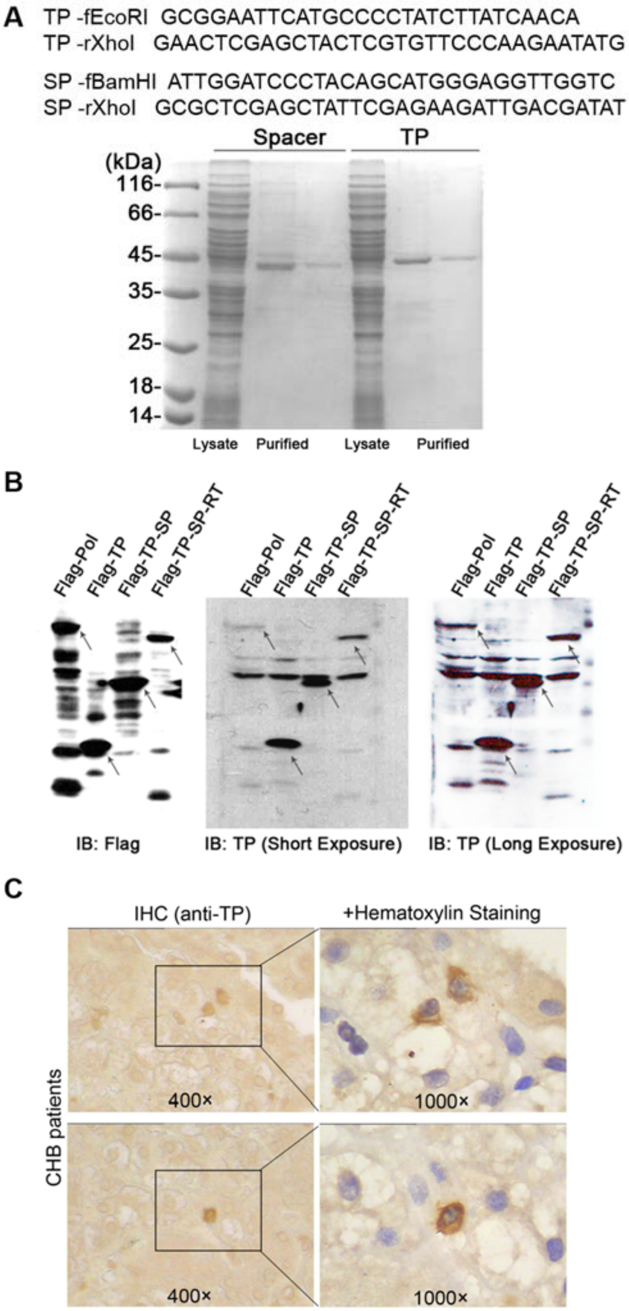

Fig S2. Preparation of the antibodies against TP and Spacer regions of viral polymerase. (A) Detection of the purified GST-Spacer and GST-TP proteins expressed in the E. coli BL21 by SDS-PAGE followed by coomassie staining. (B) Huh7 cells transfected with the indicated plasmids were extracted to detect the expression of full length- and truncated-Pol by western blotting with anti-Flag Abs or anti-TP Abs. (C) Detection of HBV proteins expression in liver biopsies from CHB patients by immunohistochemistry with anti-TP antibodies.

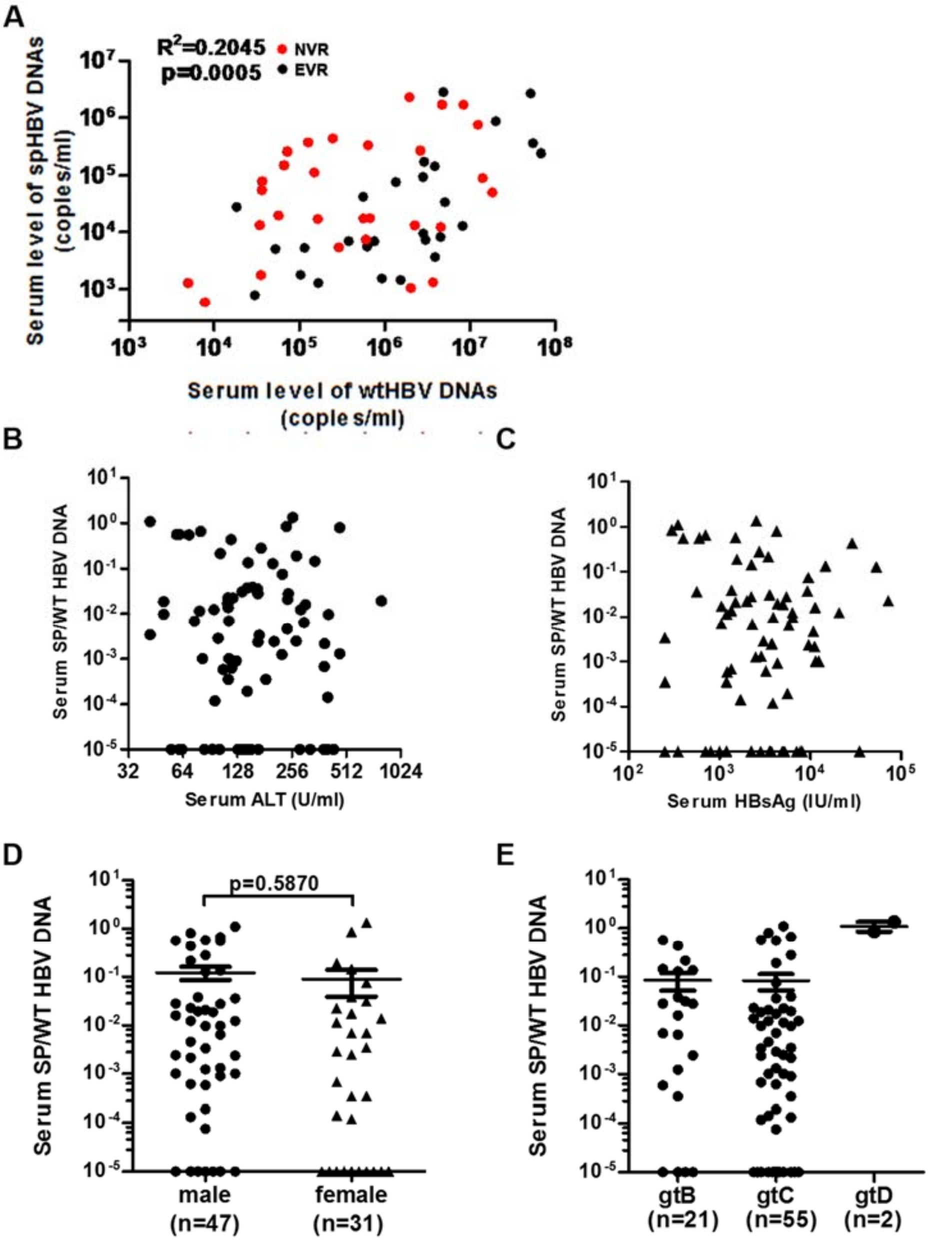

Fig S3. The correlation between spHBV variants and HBsAg, ALTs, genders or viral genotypes. (A) The level of spHBV and wtHBV DNAs in sera from the NVRs and EVRs before IFN therapy were quantified by qPCR. The relationship between spHBV DNAs and wtHBV DNAs in samples with spHBV>100 copies/ml was analyzed using Spearman's correlation. The correlation between the proportions of spHBV and the level of ALT (B) or HBsAg (C) in the sera from the 82 patients before IFN therapy. The ratio of spHBV to wtHBV DNAs in the serum samples was compared between males and females (D) (Student's t-tests) or among different genotypes (E).

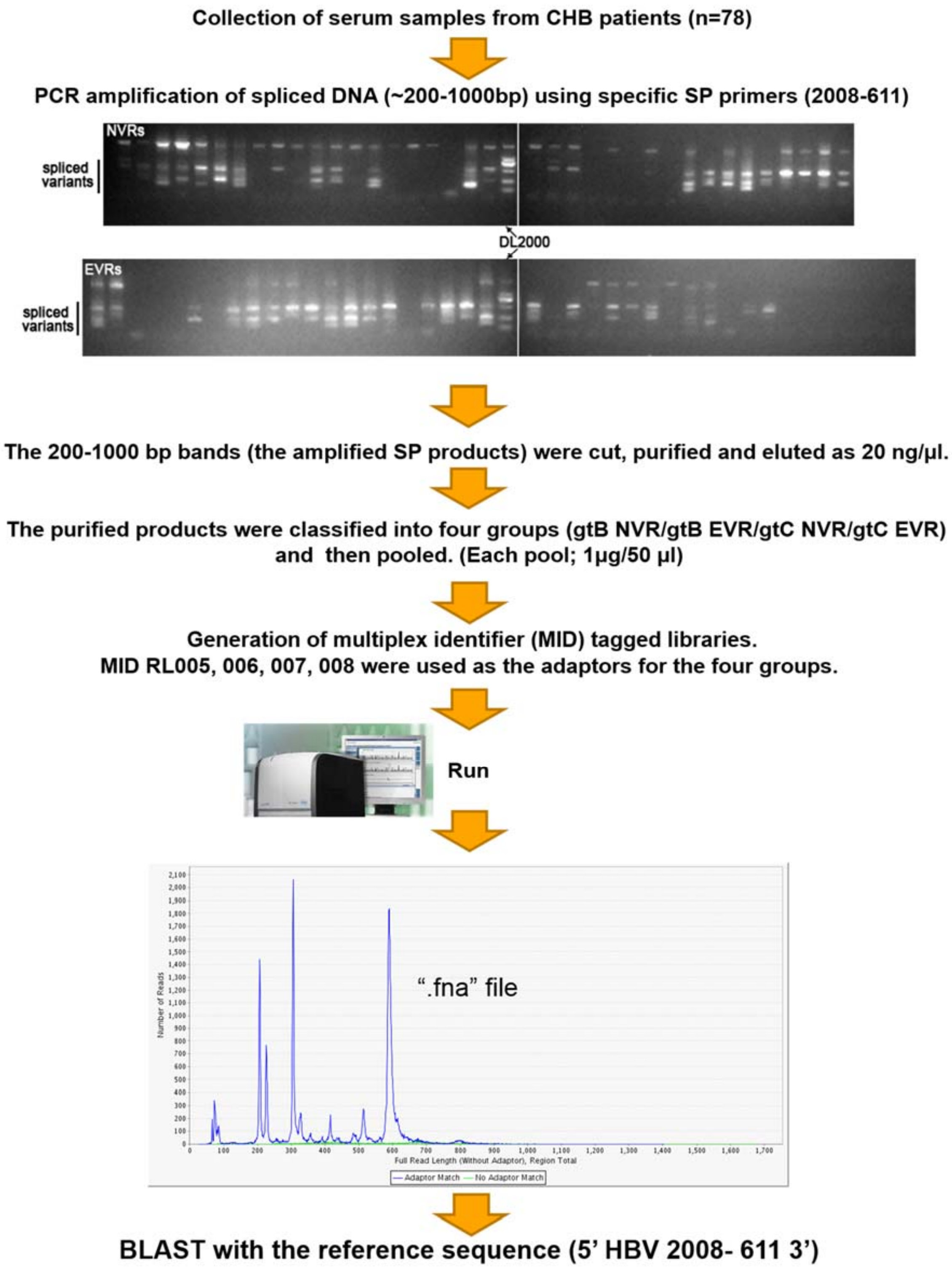

Fig S4. Work Flow of the 454 sequencing analysis of the spHBV variants in the sera of CHB patients.

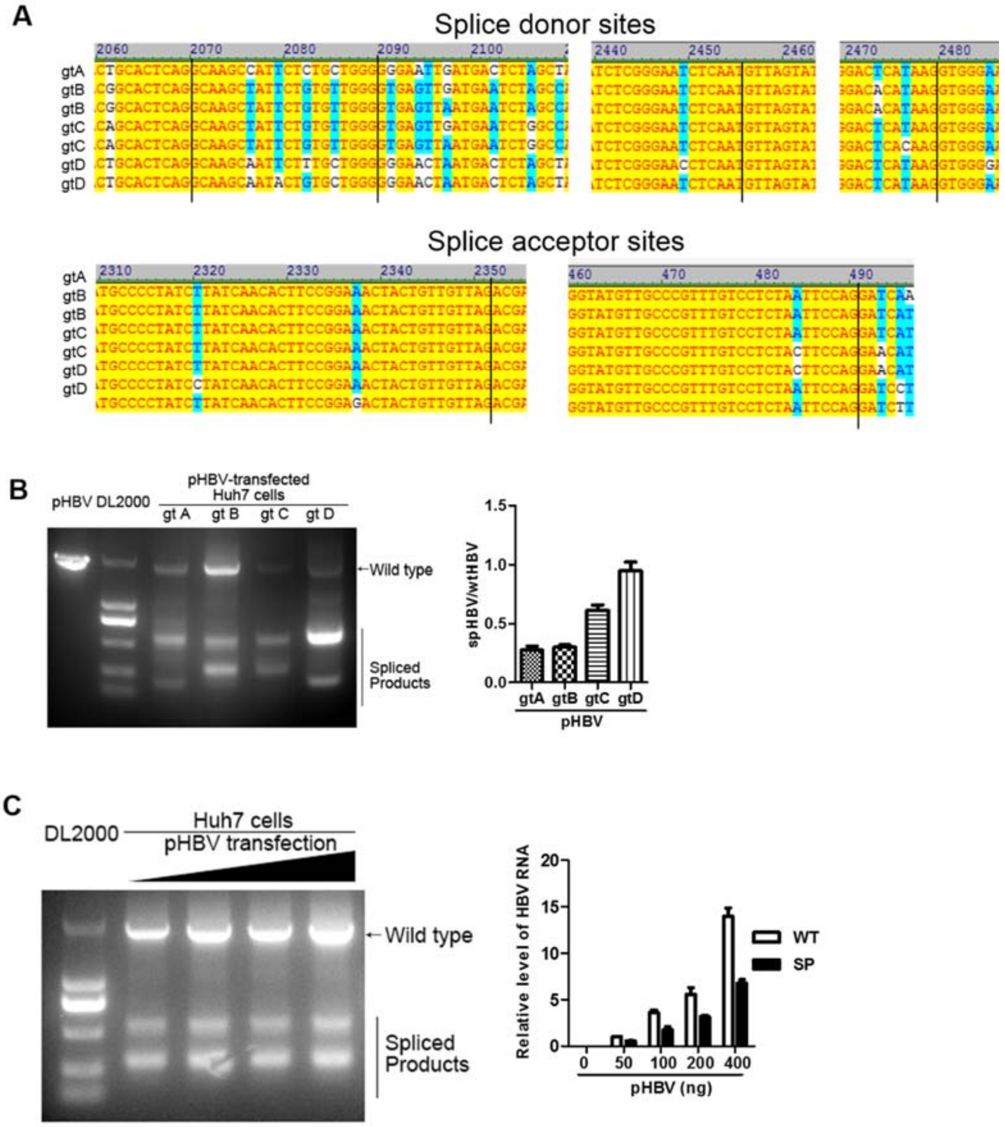

Fig S5. HBV splicing is associated with the viral genotypes. (A) The sequence alignment of the donor and acceptor sites among different HBV genotypes. (B) The cDNAs reverse-transcripted from the total RNAs extracted from Huh7 cells transfected with HBV constructs with different genotypes were analyzed by PCR followed by agarose gel electrophoresis (the left panel) and realtime PCR (the right panel). (C) The cDNAs reverse-transcripted from the total RNAs extracted from Huh7 cells transfected with different amounts of pHBV1.3 were analyzed by PCR followed by agarose gel electrophoresis (the left panel) and realtime PCR (the right panel). The data showed that the intracellular level of both the wt- and sp-HBV RNAs closely associated with the amounts of transfected plasmids, but there was no significant difference on the ratio of spHBV to wtHBV RNAs among cells transfected with different amounts of pHBV1.3.

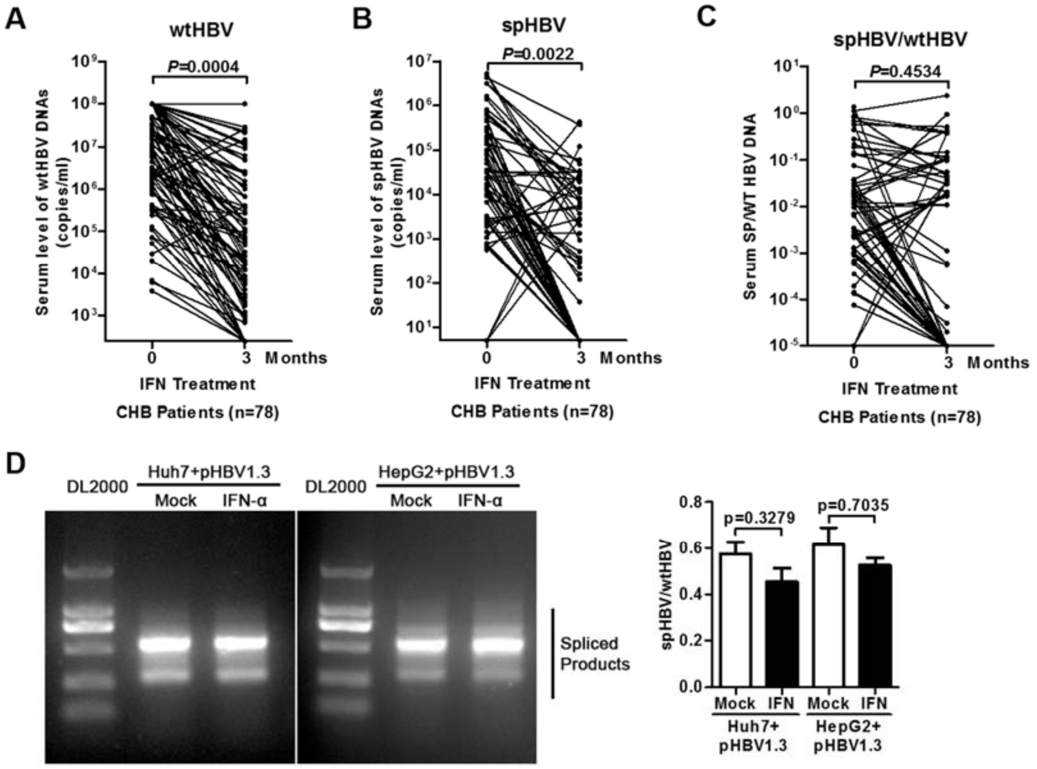

Fig S6. Effect of IFN treatment on the expression of the spHBV variants. Comparison of the absolute amount of wtHBV(A), spHBV(B), and the ratios of spHBV/wtHBV(C) DNA between paired samples from CHB patients before and after 3 months of IFN therapy. The cDNAs reverse-transcripted from the total RNAs extracted from mock- or IFN-α treated Huh7 or HepG2 cells transfected with pHBV1.3 were analyzed by PCR followed by agarose gel electrophoresis (the upper panel) and realtime PCR (the lower panel). The data, which represents results of three independent experiments performed in triplicates, was analyzed using Student's t-tests (D). To determine whether IFN treatment could affect HBV splicing, we compared the change of the amounts of spHBV and wtHBV in CHB patients following IFN therapy. Twelve weeks of IFN therapy resulted in varying degrees of reduction of both spHBV DNA level and wtHBV DNA level, and decreased the total detection rate of the serum spHBV, particularly in EVRs. Although the absolute amounts of spHBV and wtHBV decreased, the spHBV/wtHBV ratio was not much affected by IFN treatments, in both the NVRs and EVRs group. Similar results were obtained in replication-competent HBV constructs-transfected Huh7 and HepG2 cells, suggesting that IFN could globally reduce the viral DNA, including the spHBV DNA and wtHBV DNA, but did not much affect the proportions of spHBV variants among the total detected HBV genomes.

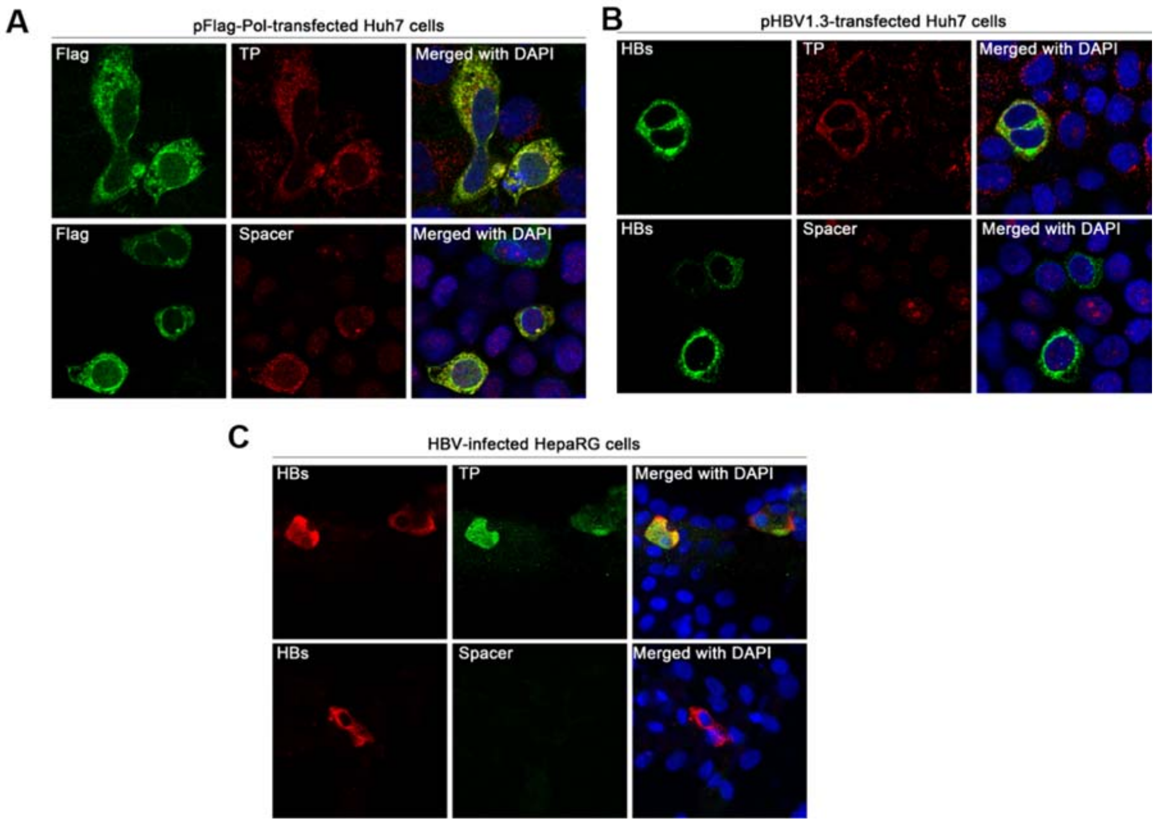

Fig S7. Detection of viral proteins expression using immunofluorescence with anti-TP and anti-SP antibodies. (A, B) Huh7 cells transfected with pFlag-Pol or pHBV1.3 were fixed, immunofluorescence stained with indicated antibodies, and analyzed using a confocal laser scanning microscope. (C) Ten days post HBV infection of the HepaRG cells, immunofluorescence assay was performed with indicated antibodies.

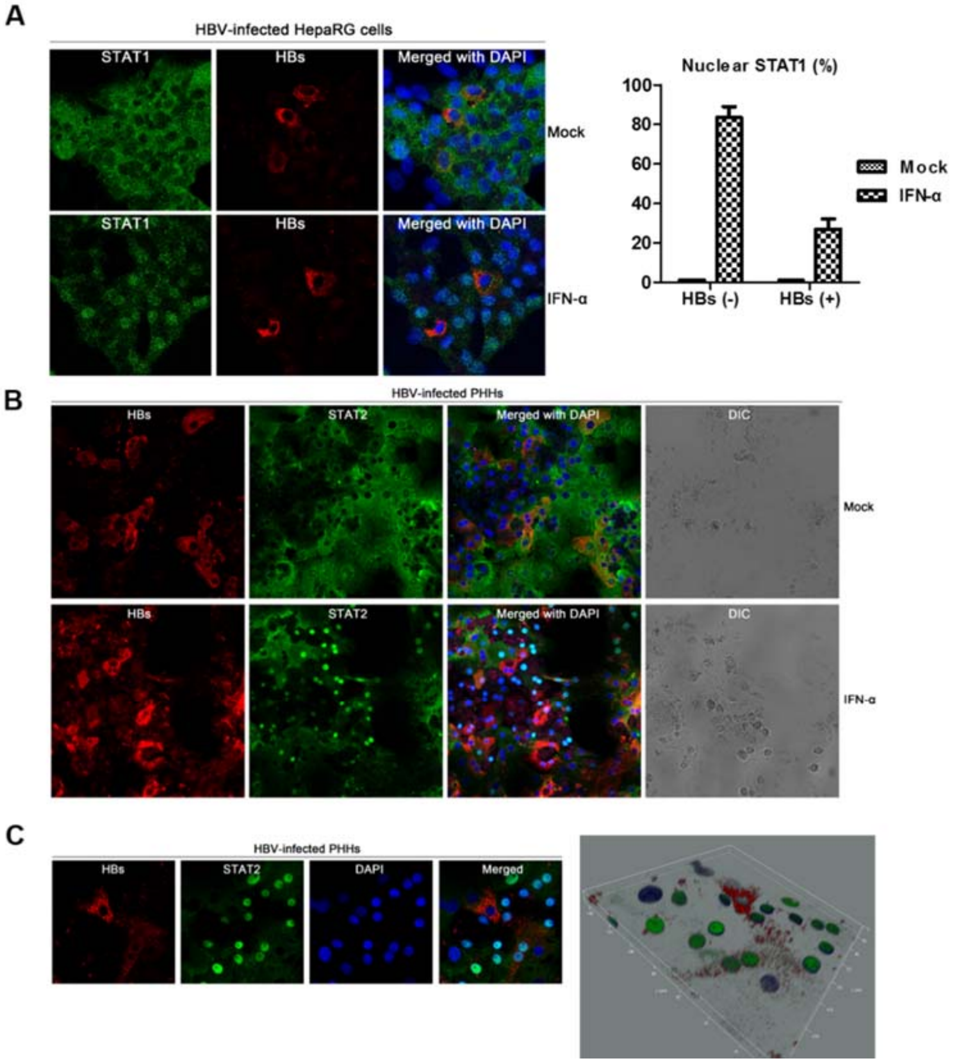

Fig S8. Impaired IFN-induced STAT1/2 nuclear accumulation in HepaRG cells and HBV-infected PHHs. (A) HepaRG cells plated in the chamber coverglass slides were treated with pooled sera from CHB patients overnight. 10 days post HBV infection, the cells were mock-treated or treated with IFN-α for 0.5 h, followed by the immunofluorescence assay with antibodies against HBs and STAT1 (the left panel). The percentage of cells with nuclear STAT1 from three independent fields covering approximately 100 HBs negative and 30 HBs positive cells was calculated (mean±SD) (the right panel). The experiments were performed three independent times in triplicates. (B) PHH cells were plated in the collagen I-pre-coated chamber coverglass slides. 10 days post HBV infection, the cells were treated with or without IFN-α for 0.5 hours, followed by the immunofluorescence assay with antibodies against HBs and STAT2. (C) The PHH cells with HBV viral protein expression showed an impaired STAT2 nuclear-translocation upon IFN treatment compared to those cells without HBV. The three-dimensional view of one of the representative field of IFN-treated PHHs were obtained by reconstruction of z-series sectioned fluorescent images using Leica Confocal Software.

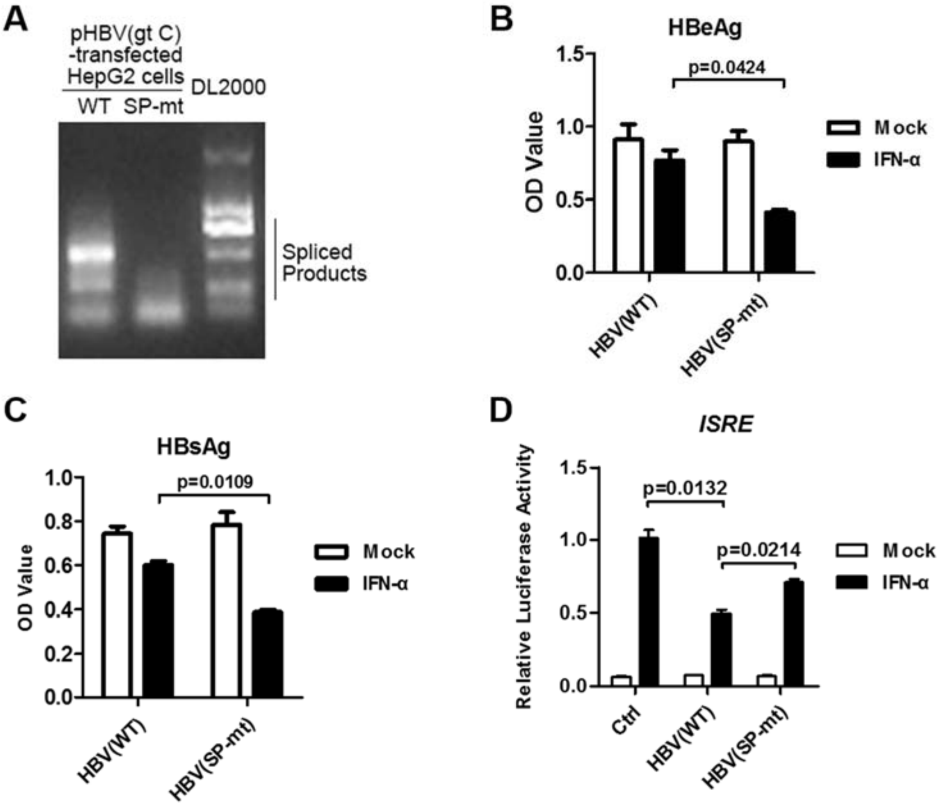

Fig S9. Mutation of the common splice sites of HBV impaired its anti-IFN activities in HepG2 cells.

The cDNAs reverse-transcripted from the total RNAs extracted from pHBV1.3- or pHBV1.3-SP-mt-transfected HepG2 cells were analyzed by PCR followed by agarose gel electrophoresis (A). The level of HBeAg and HBsAg in culture supernatants 48 h post transfection was analyzed by ELISA (B, C). HepG2 cells transfected with the indicated plasmids were mock- or IFN-α–treated for 12 hours and then harvested for luciferase reporter assay (D). The data was analyzed using Student's t-tests.

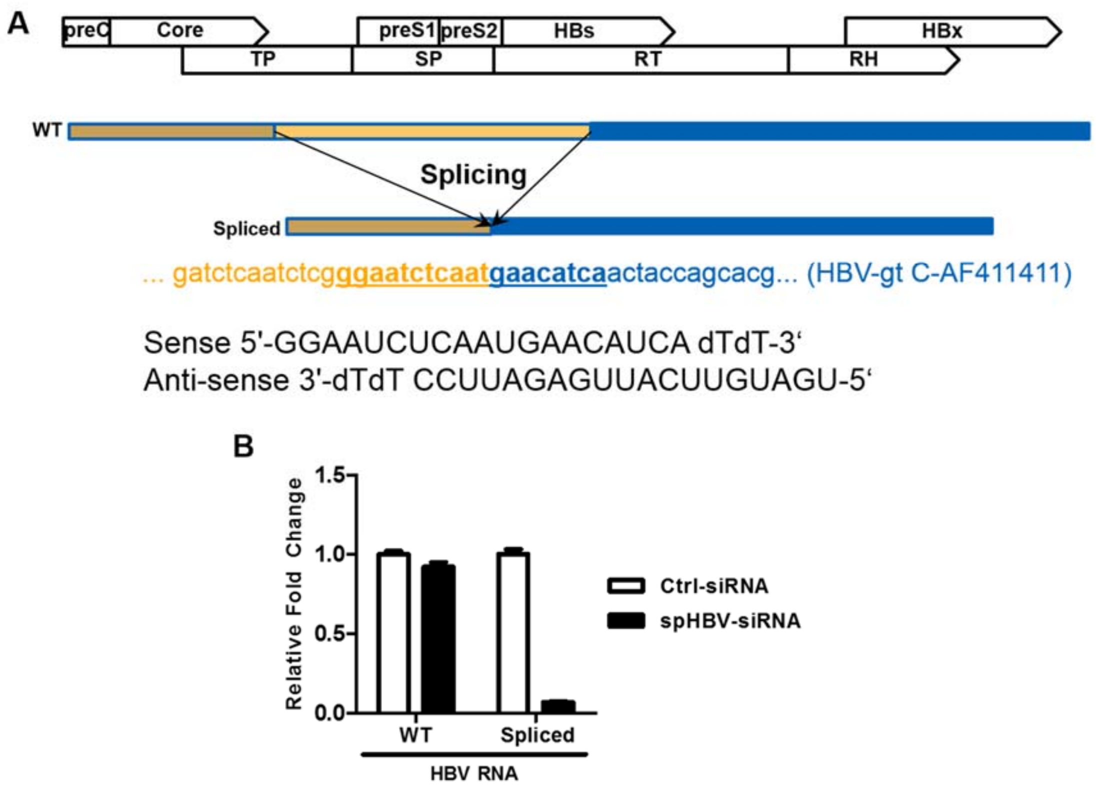

Fig S10. siRNA specifically targeting the major spHBV RNAs

(A) Schematic of the designed siRNA that specifically targets the major spliced HBV RNAs. (B) Huh7 cells were co-transfected with pHBV and control siRNAs or siRNAs targeting spHBV RNAs. 48 h post transfection, the RNAs were extracted for real-time PCR using indicated primers to determine the level of wildtype and spliced HBV RNAs.

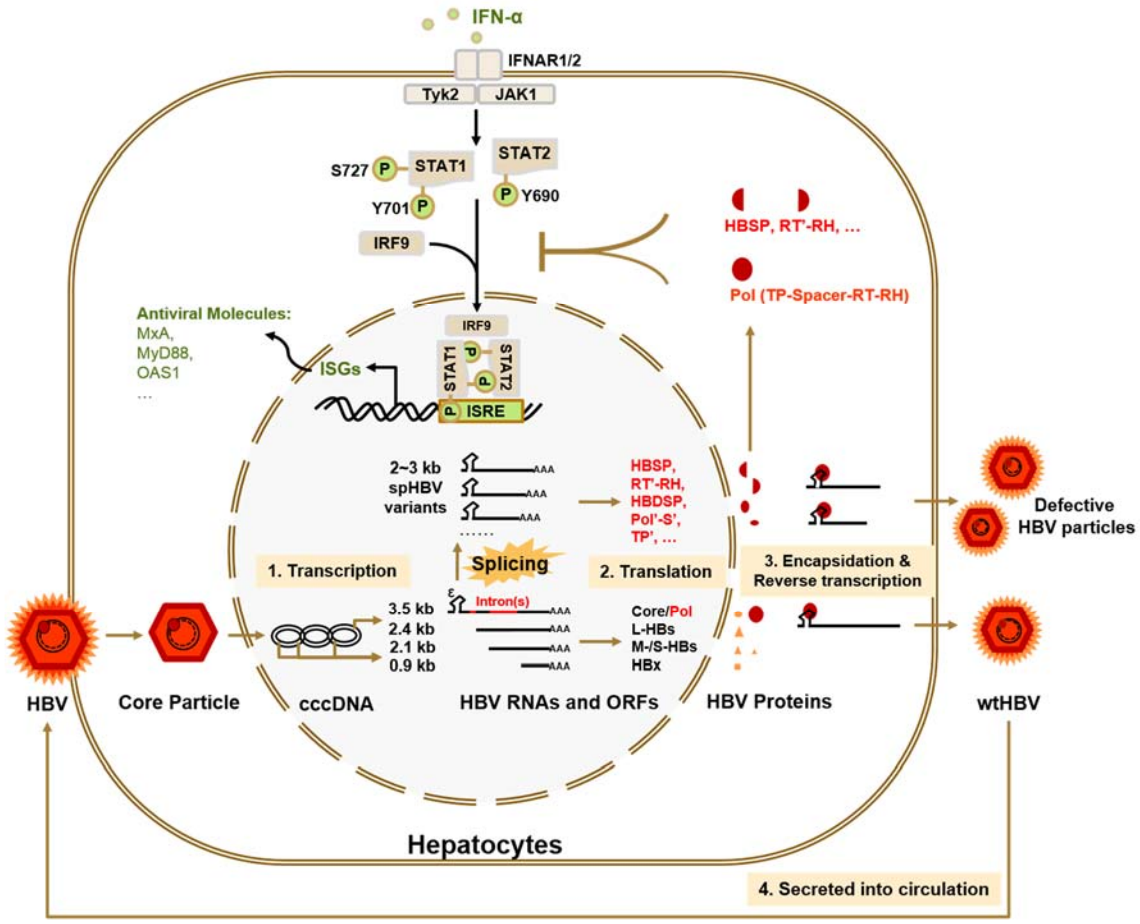

Fig S11. Model of HBV splicing-generated products-mediated inhibition on IFN signaling.

Table S1. Species and frequency of HBV spliced variants detected in the sera from CHB patients.

Types of HBV spliced variants	Exon Counts	3' splice acceptor sites	5' splice donor sites	Frequencies of HBV spliced variants (%)			
				NVRs		EVRs	
				gtB	gtC	gtB	gtC
SP1	2	488/489	2447/2448	43.59	48.60	40.96	40.25
SP2	3	2349/2350, 488/489	2067/2068, 2447/2448	17.18	17.62	23.13	23.70
SP3	2	488/489	2067/2068	9.95	7.61	9.79	9.90
SP4	3	2349/2350, 488/489	2087/2088, 2447/2448	7.99	7.52	7.69	7.15
SP5	2	488/489	2087/2088	9.84	5.42	5.66	4.68
SP6	2	488/489	2471/2472	0.42	0.26	0.11	0.25
SP7	3	2936/2937, 488/489	2447/2448, 3020/3021	0.03	0.02	0.01	0.02
SP8	4	2349/2350,2936/2937, 488/489	2067/2068, 2447/2448, 3020/3021	6.13	6.51	7.30	7.73
SP9	2	281/282	2447/2448	0.00	0.00	0.00	0.00
SP10	3	2349/2350, 281/282	2067/2068, 2447/2448	0.72	0.92	0.80	0.46
SP11	2	281/282	2471/2472	0.00	0.00	0.00	0.00
SP12	3	2235/2236, 281/282	2067/2068, 2447/2448	0.02	0.05	0.02	0.03
SP13	2	488/489	3020/3021	0.00	0.00	0.00	0.00
SP14	2	2936/2937	2447/2448	0.00	0.00	0.00	0.00
SP15*	3	2235/2236, 488/489	2067/2068, 2447/2448	1.28	1.07	1.19	1.39
SP16*	3	2349/2350, 488/489	2067/2068, 2471/2472	1.38	2.61	2.01	3.19
SP17*	2	281/282	2067/2068	1.47	1.80	1.33	1.25

Table S2. The clinical information and the level of spHBV/wtHBV of the 82 CHB patients enrolled in this study

Patient No.	Gender	Before treatment				Genotype	Serum HBV DNA (copies/ml)			Serum SP/WT HBV DNA	
		Age	HBs (U/ml	ALT(IU/m	HBeAg		0 week *	12 weeks **	Fold Reduction	0 week *	12 weeks **
1	Female	40	4900	55	+	C	2.31E+07	2.36E+06	9.788135593	<0.00001	<0.00001
2	Female	28	1350	155	+	C	1.54E+07	1.25E+06	12.32	0.038938355	<0.00001
3	Male	21	5450	166	+	B	3.36E+07	1.55E+06	21.67741935	0.027930749	0.055616892
4	Male	32	5000	50	-	C	4.72E+07	6.16E+06	7.662337662	0.018689008	0.000595133
5	Male	38	2850	141	+	C	4.99E+07	4.98E+06	10.02008032	<0.00001	0.036406297
6	Male	36	20802	95	+	C	4.86E+07	9.11E+06	5.334796926	0.012340135	0.0110562
7	Male	29	9230	145	+	B	1.07E+07	6.58E+05	16.26139818	0.037873477	0.433619142
8	Male	26	700	80	+	C	4.44E+06	4.75E+04	93.47368421	0.667265235	0.375789702
9	Female	28	3550	136	+	B	2.00E+07	2.60E+06	7.692307692	0.031206732	0.103377863
10	Male	30	1000	93	-	B	3.19E+06	2.93E+05	10.88737201	<0.00001	<0.00001
11	Male	28	4250	472	+	C	2.25E+06	3.86E+05	5.829015544	0.803573363	0.403320496
12	Female	27	1550	272	+	C	2.56E+06	1.59E+05	16.10062893	0.192109425	0.118448576
13	Female	24	1050	115	+	C	7.75E+06	1.77E+05	43.78531073	0.006963361	0.017247128
14	Female	29	2250	343	-	B	1.75E+06	1.46E+07	0.119863014	0.144987527	0.030416512
15	Male	25	2750	173	+	C	2.01E+06	2.66E+07	0.07556391	0.282632277	0.113126028
16	Female	39	2550	259	+	D	3.13E+05	7.41E+03	42.24021592	1.350975885	<0.00001
17	Male	38	250	63	+	B	7.51E+05	7.43E+04	10.1076716	<0.00001	<0.00001
18	Female	37	2250	61	+	C	4.20E+05	2.61E+04	16.09195402	<0.00001	<0.00001
19	Female	21	9500	227	+	C	3.40E+05	1.96E+04	17.34693878	0.07576458	0.020827958
20	Female	28	1200	79	+	C	3.57E+05	6.56E+04	5.442073171	0.011509732	0.929161323
21	Male	46	53122	201	+	B	2.44E+05	9.51E+04	2.565720294	0.129049604	0.147931381
22	Female	28	250	152	+	C	3.43E+05	2.68E+05	1.279850746	<0.00001	<0.00001
23	Male	36	3700	102	+	C	2.01E+04	1.03E+03	19.51456311	0.000132566	<0.00001
24	Female	28	300	239	+	D	7.17E+04	1.09E+03	65.77981651	0.855595931	0.046049603
25	Male	28	400	69	+	C	7.13E+04	1.22E+04	5.844262295	0.559547509	0.50417659
26	Male	22	350	42	+	C	2.94E+04	4.06E+05	0.072413793	1.10190465	2.36034609
27	Male	36	1500	61	+	C	6.17E+03	6.90E+02	8.942028986	0.573157051	<0.00001
28	Male	38	5600	145	+	C	1.46E+07	2.38E+06	6.134453782	0.000193417	0.034078367
29	Male	29	14700	147	+	B	1.00E+08	2.27E+06	44.05286344	0.135246876	0.041569433
30	Male	39	9552	166	+	C	1.00E+08	1.11E+07	9.009009009	0.002387971	<0.00001
31	Male	28	11150	389	+	C	1.00E+08	4.84E+06	20.66115702	0.002174653	0.016839571
32	Male	22	28850	118	+	B	1.00E+08	2.15E+07	4.651162791	0.440433932	0.09654411
33	Male	27	6350	50	+	C	1.00E+08	2.28E+07	4.385964912	0.009834045	2.0039E-05
34	Male	32	10800	241	+	C	1.00E+08	1.23E+07	8.130081301	0.004626072	0.000651476
35	Male	26	12400	115	+	C	1.00E+08	2.82E+07	3.546099291	0.001013808	3.08829E-05
36	Male	28	72281	114	+	C	1.00E+08	1.00E+08	1	0.02279193	0.001120481
37	Male	26	11450	82	+	C	1.00E+08	2.18E+07	4.587155963	0.001018033	<0.00001
38	Male	24	11350	305	+	B	1.00E+08	6.74E+05	148.3679525	0.016090284	0.101320541
39	Female	55	3550	403	+	C	1.00E+08	1.00E+06	100	<0.00001	<0.00001
40	Female	18	5212	385	+	C	3.42E+05	1.01E+03	338.6138614	<0.00001	<0.00001
41	Male	40	561	165	+	C	8.53E+05	7.22E+03	118.1440443	0.036088069	<0.00001
42	Female	28	7725	152	+	C	2.22E+06	2.04E+04	108.8235294	<0.00001	<0.00001
43	Female	32	250	184	+	C	2.93E+06	1.26E+04	232.5396825	0.000352685	<0.00001
44	Male	27	250	169	+	C	2.12E+07	1.15E+05	184.3478261	0.003353	0.000552911
45	Female	34	250	42	+	C	2.74E+07	1.84E+05	148.9130435	0.003452043	<0.00001
46	Male	39	5800	300	+	B	2.58E+07	250	103200	0.006443214	<0.00001
47	Female	33	1350	387	+	C	1.00E+08	5.53E+03	18083.18264	0.000681578	<0.00001
48	Female	22	2000	121	+	C	3.03E+07	6.72E+03	4508.928571	0.022436593	<0.00001
49	Male	32	1216	107	+	B	1.00E+08	1.23E+03	81300.81301	0.000586872	<0.00001
50	Female	31	34800	129	+	C	4.39E+07	2.03E+03	21625.61576	<0.00001	<0.00001
51	Male	25	3450	103	+	B	1.81E+07	4.20E+03	4309.52381	0.217939567	0.053598421
52	Male	30	3900	408	+	C	1.00E+08	3.23E+04	3095.975232	0.009691938	0.010964611
53	Female	28	5000	284	+	C	2.70E+07	5.40E+04	500	<0.00001	<0.00001
54	Female	26	3600	143	+	C	1.18E+07	7.05E+04	167.3758865	<0.00001	0.374749805
55	Male	29	4350	126	+	C	1.00E+08	2.59E+04	3861.003861	0.000909272	<0.00001
56	Male	42	2900	471	+	C	1.00E+08	1.96E+04	5102.040816	0.00131657	<0.00001
57	Male	25	3750	204	+	B	1.00E+08	3.71E+05	269.541779	0.002439272	0.017361064
58	Male	36	4350	803	+	C	1.00E+08	1.69E+05	591.7159763	0.019419709	0.110913071
59	Female	55	3800	271	+	C	4.77E+07	1.09E+05	437.6146789	0.002484204	<0.00001
60	Male	33	3250	119	-	C	1.24E+06	7.88E+02	1573.604061	0.000618331	0.020474942
61	Male	25	6350	287	+	C	6.83E+06	1.77E+03	3858.757062	0.012410181	6.97071E-05
62	Male	49	2500	226	+	B	8.12E+06	1.71E+03	4748.538012	0.00124728	0.017762782
63	Female	29	1200	114	+	B	1.34E+06	250	5360	0.000352359	<0.00001
64	Female	31	3850	96	+	C	9.53E+06	8.62E+03	1105.568445	0.000118842	<0.00001
65	Female	38	1350	114	+	C	7.29E+06	3.22E+03	2263.975155	0.013888166	<0.00001
66	Female	20	2300	74	+	B	5.74E+06	250	22960	0.006904083	<0.00001
67	Male	33	1500	243	+	C	2.13E+06	2.17E+03	981.5668203	0.02095344	<0.00001
68	Male	32	2250	245	-	B	1.03E+06	250	4120	0.028001874	<0.00001
69	Male	31	8100	84	+	C	1.60E+06	5.59E+03	286.2254025	<0.00001	<0.00001
70	Male	28	1200	436	+	C	3.28E+06	1.84E+04	178.2608696	<0.00001	<0.00001
71	Female	26	1700	405	+	C	3.28E+06	1.64E+04	200	0.000142574	<0.00001
72	Female	24	800	167	+	B	1.30E+05	9.65E+02	134.7150259	<0.00001	0.038473243
73	Male	26	350	397	+	B	4.18E+05	250	1672	<0.00001	<0.00001
74	Female	21	1050	113	+	C	1.11E+05	250	444	0.017305012	<0.00001
75	Female	25	3050	100.00	+	C	9.51E+05	2.71E+03	350.9225092	0.002878105	<0.00001
76	Male	31	7040	324	-	C	5.18E+04	250	207.2	<0.00001	<0.00001
77	Male	25	700	136	-	C	6.84E+04	250	273.6	<0.00001	<0.00001
78	Male	46	600	59	-	B	3.82E+04	250	152.8	0.568407525	<0.00001

0 week	SP dtection rate (positive/total)	AVG of SP/WT	Two-tailed t-test
NVRs（<2 log）	31/37	0.203832576	p=0.00215
EVRs（>2 log）	29/41	0.024805674	
**12 weeks**	**SP dtection rate (positive/total)**	**AVG of SP/WT**	
NVRs（<2 log）	26/37	0.159418305	p=0.03611
EVRs（>2 log）	11/41	0.018208319	

* Before IFN treatment; ** After 12 weeks of IFN treatment;

“SP-positive” was defined as spHBV positive and spHBV/wtHBV >0.00001;
The viral load was determined using an HBV DNA quantitative real-time PCR kit (Qiagen) and HBV genotyping was performed as previously reported (1).

Table S3. Regression analysis of the factors related to the prognosis of IFN responses

HBs (U/ml)	SP/WT DNA	*Fold Reduction	Gender	Age	ALT(IU/ml	HBs(Log)	SP/WT DNA (Log)	Fold Reduction (Log)	
4900	0.00001	9.788135593	2	40	55	3.69019608	-5	0.990699977	1
1350	0.038938355	12.32	2	28	155	3.130333768	-1.409622394	1.090610708	1
5450	0.027930749	21.67741935	1	21	166	3.736396502	-1.553917418	1.336007579	1
5000	0.018689008	7.662337662	1	32	50	3.698970004	-1.728413739	0.884361286	1
2850	0.00001	10.02008032	1	38	141	3.45484486	-5	1.000871203	1
20802	0.012340135	5.334796926	1	36	95	4.318105092	-1.908680077	0.727117892	1
9230	0.037873477	16.26139818	1	29	145	3.965201701	-1.421664827	1.211157884	1
700	0.667265235	93.47368421	1	26	80	2.84509804	-0.175701502	1.97068936	1
3550	0.031206732	7.692307692	2	28	136	3.550228353	-1.505751708	0.886056648	1
1000	0.00001	10.88737201	1	30	93	3	-5	1.036923063	1
4250	0.803573363	5.829015544	1	28	472	3.62838893	-0.094974468	0.765595213	1
1550	0.192109425	16.10062893	2	27	272	3.190331698	-0.716451328	1.206842841	1
1050	0.006963361	43.78531073	2	24	115	3.021189299	-2.157181059	1.641328436	1
2250	0.144987527	0.119863014	2	29	343	3.352182518	-0.838669357	-0.921314807	1
2750	0.282632277	0.07556391	1	25	173	3.439332694	-0.548778242	-1.121685579	1
2550	1.350975885	42.24021592	2	39	259	3.40654018	0.130647597	1.62572613	1
250	0.00001	10.1076716	1	38	63	2.397940009	-5	1.004651123	1
2250	0.00001	16.09195402	2	37	61	3.352182518	-5	1.206608783	1
9500	0.07576458	17.34693878	2	21	227	3.977723605	-1.120533777	1.239222846	1
1200	0.011509732	5.442073171	2	28	79	3.079181246	-1.938934772	0.735764377	1
53122	0.129049604	2.565720294	1	46	201	4.725274418	-0.889243324	0.409209309	1
250	0.00001	1.279850746	2	28	152	2.397940009	-5	0.107159326	1
1500	7.57706E-05	19.51456311	1	36	102	3.568201724	-3.87756753	1.290358833	1
3700	0.00001	65.77981651	2	28	239	2.477121255	-0.06773129	1.818092658	1
1150	0.00001	5.844262295	1	28	69	2.602059991	-0.252163033	0.766729699	1
300	0.855595931	0.072413793	1	22	42	2.544068044	0.042144016	-1.140178703	1
400	0.559547509	8.942028986	1	36	61	3.176091259	-0.241726361	0.951436073	1
350	1.10190465	6.134453782	1	38	145	3.748188027	-3.71350616	0.787775899	1
1500	0.573157051	44.05286344	1	29	147	4.167317335	-0.868872759	1.643974143	1
5600	0.000193417	9.009009009	1	39	166	3.980094314	-2.621970987	0.954677021	1
15150	0.000132566	20.66115702	1	28	389	4.047274867	-2.662610105	1.315154638	1
14700	0.135246876	4.651162791	1	22	118	4.460145817	-0.356119229	0.66756154	1
9552	0.002387971	4.385964912	1	27	50	3.802773725	-2.007267791	0.642065153	1
11150	0.002174653	8.130081301	1	32	241	4.033423755	-2.334787654	0.910094889	1
28850	0.440433932	3.546099291	1	26	115	4.093421685	-2.994044286	0.549750892	1
6350	0.009834045	1	1	28	114	4.859024152	-1.642218892	0	1
10800	0.004626072	4.587155963	1	26	82	4.058805487	-2.99223795	0.661543506	1
12400	0.001013808	148.3679525	1	24	305	4.054995862	-1.79343629	2.171340103	2
72281	0.02279193	100	2	55	403	3.550228353	-5	2	2
11450	0.001018033	338.6138614	2	18	385	3.717004407	-5	2.529704732	2
11350	0.016090284	118.1440443	1	40	165	2.748962861	-1.442636355	2.072411834	2
3550	0.00001	108.8235294	2	28	152	3.887898488	-5	2.036722807	2
5212	0.00001	232.5396825	2	32	184	2.397940009	-3.452613076	2.366497075	2
561	0.036088069	184.3478261	1	27	169	2.397940009	-2.474566454	2.265638021	2
7725	0.00001	148.9130435	2	34	42	2.397940009	-2.461923824	2.17293274	2
250	0.000352685	103200	1	39	300	3.763427994	-2.19089742	5.013679697	2
250	0.003353	18083.18264	2	33	387	3.130333768	-3.166484492	4.257274869	2
250	0.003452043	4508.928571	2	22	121	3.301029996	-1.649043083	3.654073355	2
5800	0.006443214	81300.81301	1	32	107	3.084933575	-3.231456325	4.910094889	2
1350	0.000681578	21625.61576	2	31	129	4.541579244	-5	4.334968482	2
2000	0.022436593	4309.52381	1	25	103	3.537819095	-0.661663917	3.634429284	2
1216	0.000586872	3095.975232	1	30	408	3.591064607	-2.013589393	3.490797478	2

Regression Analysis

OVERALL FIT

Multiple R	0.3421102
R Square	0.1170394
Adjusted R Squ	0.0557227
Standard Erro	1.2941637
Observations	78

ANOVA — Alpha 0.05

	df	*SS*	*MS*	*F*	*p-value*	*sig*
Regression	5	15.98459	3.196918	1.9087678	0.1034007	no
Residual	72	120.58989	1.6748596			
Total	77	136.57448				

	coeff	*std err*	*t stat*	*p-value*	*lower*	*upper*
Intercept	1.3641042	1.2830054	1.06321	0.2912397	-1.19352	3.9217288
Gender	0.1692558	0.3134792	0.5399265	0.5909138	-0.455654	0.7941652
Age	0.0084977	0.0200945	0.4228839	0.6736404	-0.03156	0.0485554
ALT(IU/ml)	0.0021851	0.0011098	1.9689618	0.0528068	-2.72E-05	0.0043975
HBs(Log)	-0.217254	0.2719143	-0.79898	0.4269301	-0.759305	0.3247972
SP/WT DNA (L	-0.167781	0.0923704	-1.816397	0.073472	-0.351918	0.0163557

34800	0.00001	500	2	28	284	3.698970004	-5	2.698970004	2
3450	0.217939567	167.3758865	2	26	143	3.556302501	-5	2.22369289	2
3900	0.009691938	3861.003861	1	29	126	3.638489257	-3.041306239	3.586700236	2
5000	0.00001	5102.040816	1	42	471	3.462397998	-2.880556102	3.707743929	2
3600	0.00001	269.541779	1	25	204	3.574031268	-2.612739686	2.43062609	2
4350	0.000909272	591.7159763	1	36	803	3.638489257	-1.711757283	2.772113295	2
2900	0.00131657	437.6146789	2	55	271	3.579783597	-2.604812707	2.641091881	2
3750	0.002439272	1573.604061	1	33	119	3.511883361	-3.208779131	3.196895468	2
4350	0.019419709	3858.757062	1	25	287	3.802773725	-1.906221867	3.586447437	2
3800	0.002484204	4748.538012	1	49	226	3.397940009	-2.904036175	3.676559919	2
3250	0.000618331	5360	2	29	114	3.079181246	-3.453014229	3.72916479	2
6350	0.012410181	1105.568445	2	31	96	3.58546073	-3.925029501	3.043585635	2
2500	0.00124728	2263.975155	2	38	114	3.130333768	-1.857355096	3.354871657	2
1200	0.000352359	22960	2	20	74	3.361727836	-2.16089402	4.360971884	2
3850	0.000118842	981.5668203	1	33	243	3.176091259	-1.678744662	2.99191987	2
1350	0.013888166	4120	1	32	245	3.352182518	-1.552812908	3.614897216	2
2300	0.006904083	286.2254025	1	31	84	3.908485019	-5	2.456708175	2
1500	0.02095344	178.2608696	1	28	436	3.079181246	-5	2.251056021	2
2250	0.028001874	200	2	26	405	3.230448921	-3.845959519	2.301029996	2
8100	0.00001	134.7150259	2	24	167	2.903089987	-5	2.129416039	2
1200	0.00001	1672	1	26	397	2.544068044	-5	3.223236273	2
1700	0.000142574	444	2	21	113	3.021189299	-1.761828087	2.64738297	2
800	0.00001	350.9225092	2	25	100.00	3.484299839	-2.540893326	2.545211226	2
350	0.00001	207.2	1	31	324	3.847572659	-5	2.316389751	2
1050	0.017305012	273.6	1	25	136	2.84509804	-5	2.437116093	2
3050	0.002878105	152.8	1	46	59	2.77815125	-0.245340181	2.184123354	2
7040	0.00001								
700	0.00001								
600	0.568407525								

2: Female
1: Male

* Fold Reduction of viral DNA after 3 months therapy

We have applied the multiple linear regression models (http://www.real-statistics.com/multiple-regression/) to determine whether independent variables including gender, age, ALT, HBsAg (Log), spHBV/wtHBV (Log) are making a significant contribution to the fold of viral DNA reduction after IFN therapy. Results showed that ALT and the ratio of sp/wt HBV tend to be associated with the responses to IFN treatment. The ratio of sp/wt HBV was statistically associated with the responses to IFN treatment when the patients with undetectable spHBV DNAs were excluded.

Gender	Age	ALT(IU/ml)	HBsAg(Log	SP/WT DNA （Log）	old Reduction of viral DNA （Log）
2	28	155	3.1303338	-1.409622394	1.090610708
1	21	166	3.7363965	-1.553917418	1.336007579
1	32	50	3.69897	-1.728413739	0.884361286
1	36	95	4.3181051	-1.908680077	0.727117892
1	29	145	3.9652017	-1.421664827	1.211157884
1	26	80	2.845098	-0.175701502	1.97068936
2	28	136	3.5502284	-1.505751708	0.886056648
1	28	472	3.6283889	-0.094974468	0.765595213
2	27	272	3.1903317	-0.716451328	1.206842841
2	24	115	3.0211893	-2.157181059	1.641328436
2	29	343	3.3521825	-0.838669357	-0.921314807
1	25	173	3.4393327	-0.548778242	-1.121685579
2	39	259	3.4065402	0.130647597	1.62572613
2	21	227	3.9777236	-1.120533777	1.239222846
2	28	79	3.0791812	-1.938934772	0.735764377
1	46	201	4.7252744	-0.889243324	0.409209309
2	28	239	2.4771213	-0.06773129	1.818092658
1	28	69	2.60206	-0.252163033	0.766729699
1	22	42	2.544068	0.042144016	-1.140178703
1	36	61	3.1760913	-0.241726361	0.951436073
1	38	145	3.748188	-3.71350616	0.787775899
1	29	147	4.1673173	-0.868872759	1.643974143
1	39	166	3.9800943	-2.621970987	0.954677021
1	28	389	4.0472749	-2.662610105	1.315154638
1	22	118	4.4601458	-0.356119229	0.66756154
1	27	50	3.8027737	-2.007267791	0.642065153
1	32	241	4.0334238	-2.334787654	0.910094889
1	26	115	4.0934217	-2.994044286	0.549750892
1	28	114	4.8590242	-1.642218892	0
1	26	82	4.0588055	-2.99223795	0.661543506
1	24	305	4.0549959	-1.79343629	2.171340103
1	40	165	2.7489629	-1.442636355	2.072411834
2	32	184	2.39794	-3.452613076	2.366497075
1	27	169	2.39794	-2.474566454	2.265638021
2	34	42	2.39794	-2.461923824	2.17293274
1	39	300	3.763428	-2.19089742	5.013679697
2	33	387	3.1303338	-3.166484492	4.257274869
2	22	121	3.30103	-1.649043083	3.654073355
1	32	107	3.0849336	-3.231456325	4.910094889
1	25	103	3.5378191	-0.661663917	3.634429284
1	30	408	3.5910646	-2.013589393	3.490797478
1	29	126	3.6384893	-3.041306239	3.586700236
1	42	471	3.462398	-2.880556102	3.707743929
1	25	204	3.5740313	-2.612739686	2.43062609
1	36	803	3.6384893	-1.711757283	2.772113295
2	55	271	3.5797836	-2.604812707	2.641091881
1	33	119	3.5118834	-3.208779131	3.196895468
1	25	287	3.8027737	-1.906221867	3.586447437
1	49	226	3.39794	-2.904036175	3.676559919
2	29	114	3.0791812	-3.453014229	3.72916479
2	31	96	3.5854607	-3.925029501	3.043585635
2	38	114	3.1303338	-1.857355096	3.354871657
2	20	74	3.3617278	-2.16089402	4.360971884
1	33	243	3.1760913	-1.678744662	2.99191987
1	32	245	3.3521825	-1.552812908	3.614897216
2	26	405	3.2304489	-3.845959519	2.301029996

Regression Analysis

OVERALL FIT

Multiple I	0.5466
R Square	0.2987
Adjusted R S	0.2326
Standard Er	1.258
Observatio	59

ANOVA — Alpha 0.05

	df	SS	MS	F	p-value	sig
Regressio	5	35.727	7.1454	4.5152	0.0017	yes
Residual	53	83.873	1.5825			
Total	58	119.6				

	coeff	std err	t stat	p-value	lower	upper
Intercept	2.5351	1.6067	1.5779	0.1205	-0.687	5.7577
Gender	-0.045	0.3786	-0.12	0.9049	-0.805	0.7139
Age	0.0177	0.0234	0.7562	0.4529	-0.029	0.0646
ALT(IU/n	0.0019	0.0012	1.5321	0.1315	-6E-04	0.0044
HBsAg(Lo	-0.714	0.3269	-2.183	0.0335	-1.37	-0.058
SP/WT DNA （	-0.578	0.1567	-3.689	0.0005	-0.892	-0.264

2	21	113	3.0211893	-1.761828087	2.64738297
2	25	100	3.4842998	-2.540893326	2.545211226
1	46	59	2.7781513	-0.245340181	2.184123354

SP/WT HBV DNA>0.00001

Table S4. Primers for qPCR detection of OAS-1, GAPDH, mMx1, mSTAT1 and mGAPDH

Gene	Primer Sequences(5'→3')	Length
OAS-1	F- TGCCAGACACGTGTTTCCGC<br>R- GAGGAAGACAACCAGGTCAGCG	185 bp
GAPDH	F- GGTATCGTGGAAGGACTCATGA<br>R- ATGCCAGTGAGCTTCCCGTTCAGC	188 bp
mMx1	F- GGATGTGATGCGGAACCT<br>R- AGAAGAATGCTGAAGTATGAGTGA	156 bp
mSTAT1	F- CTTCCACGACCTCCTCTC<br>R- CTTCCTTCAGACAGTTGTAGATG	167 bp
mGAPDH	F- AGTATGTCGTGGAGTCTA<br>R- CAATCTTGAGTGAGTTGTC	162 bp

References

1. Naito H, Hayashi S, Abe K. Rapid and specific genotyping system for hepatitis B virus corresponding to six major genotypes by PCR using type-specific primers. J Clin Microbiol 2001;39:362-364.
